# Artificial Intelligence-Driven Sensing of Cross-Border Trade Risks Through Declaration-to-Physical-Fact Alignment and Evidence-Grounded Question Answering

**DOI:** 10.3390/s26165272

**Published:** 2026-08-20

**Authors:** Meitong Chen, Jiayi Huang, Zilang Zhou, Zhonghao Zhang, Kele Lei, Yongxin Tang, Manzhou Li

**Affiliations:** 1 China Agricultural University, Beijing 100083, China; 2 Peking University, Beijing 100871, China; 3 Renmin University of China, Beijing 100872, China

**Keywords:** multisource hardware sensors, multimodal sensor fusion, trade anomaly detection, reliability-aware representation learning, intelligent port monitoring

## Abstract

Cross-border trade security risks are often embedded in inconsistencies among trade documents, logistics trajectories, hardware sensor states, and financial settlement activities. Existing methods primarily rely on structured declaration fields, making it difficult to verify digital declarations against actual physical processes or to generate complete evidence suitable for regulatory review. To address these challenges, TradeSense-EQA is proposed as a cross-border trade security anomaly detection and evidence-grounded English question-answering framework. Multisource sensing information, including trade documents, GPS/AIS trajectories, RFID records, electronic seal events, port weighing data, temperature and humidity measurements, vibration signals, container door states, and visual images, is jointly modeled within the framework. The reliability-aware representation module dynamically adjusts sensing-channel weights according to data missingness, sampling intervals, device health states, and communication quality. The trade-process-constrained module identifies anomalies across declaration, packing, transportation, transshipment, arrival, and customs clearance stages and generates process-consistent evidence chains. The evidence-grounded question-answering module answers English trade risk questions on the basis of verified documentary fields and sensor records, while confidence estimation and abstention mechanisms are incorporated to reduce factual hallucinations. Experimental results demonstrate that TradeSense-EQA achieved an Accuracy of 0.918, a Precision of 0.909, a Recall of 0.897, a Macro-F1 of 0.903, and a ROC-AUC of 0.958 on the cross-border trade anomaly detection task, outperforming baseline methods including XGBoost, LightGBM, TCN, Transformer, BERT, CLIP, and VisualBERT. On the English trade risk question-answering task, Exact Match, Token-level F1, BLEU, ROUGE-L, and BERTScore reached 0.782, 0.851, 0.668, 0.801, and 0.934, respectively. Ablation results further confirmed the effectiveness of hardware sensing input, reliability-aware weighting, declaration–fact alignment, process-graph reasoning, and evidence-constrained generation. The proposed framework provides a reliable, interpretable, and auditable artificial intelligence-driven sensing solution for customs supervision, port security, international logistics review, and trade-background investigation.

## 1. Introduction

Cross-border trade forms the backbone of global supply chains and international commerce [1], involving multiple stages from cargo declaration and transportation to customs clearance and financial settlement [2]. The rapid digitalization of trade and intelligent port infrastructure has led to the large-scale deployment of trade documents together with multisource sensing devices, including radio frequency identification (RFID), global positioning system (GPS), automatic identification system (AIS), electronic seals, weighing systems, environmental sensors, and visual inspection equipment [3]. These heterogeneous data provide complementary observations of both declared information and physical cargo movements, creating new opportunities for trade authenticity verification and security risk detection [4]. Unlike conventional anomalies in isolated data fields, cross-border trade risks are typically reflected by inconsistencies between declared transactions and their actual execution [5]. Fraudulent activities such as false declarations, abnormal transshipment, circular transactions, and trade-based money laundering often span multiple trade stages and cannot be reliably identified from a single information source [6]. Effective risk identification therefore requires jointly analyzing documentary records, logistics trajectories, multisource sensing observations, and financial settlement information to verify the consistency of the complete trade process [7]. Existing approaches mainly rely on expert rules, statistical models, or machine learning algorithms operating on structured declaration data [8]. Although machine learning improves nonlinear pattern modeling, current methods still lack systematic integration of documentary, logistics, sensing, and financial evidence [9], resulting in fragmented risk perception and limited capability for verifying trade authenticity across multiple process stages [10]. Consequently, developing a unified multimodal framework capable of process-aware evidence fusion and cross-source consistency verification remains an important challenge for intelligent cross-border trade risk detection.

Recent advances in deep learning have substantially promoted cross-border trade anomaly detection by enabling multimodal analysis of heterogeneous trade data [11,12]. Pretrained language models can identify semantic inconsistencies in trade documents, temporal models capture long-term dependencies in logistics and sensor sequences [13], vision models verify cargo appearance and container information, graph neural networks model enterprise and transaction relationships [14], and multimodal Transformers further integrate textual, visual, trajectory, and sensing information into unified representations [15]. These developments have significantly improved the capability of intelligent trade risk analysis [16]. Recent studies have highlighted the importance of interpretable fraud detection, multimodal reasoning, and evidence-grounded question answering [17]. For example, Xu et al. [18] combined artificial intelligence with interpretable decision support for cross-border trade fraud detection, while Chen et al. [19] demonstrated that integrating speech, vision, and textual information can substantially improve multimodal question answering.

Nevertheless, several important challenges remain. First, hardware sensing data are frequently affected by missing observations, sampling asynchrony, device drift, communication failures, and measurement noise, making conventional feature fusion vulnerable to unreliable sensors. Second, existing methods mainly emphasize multimodal feature learning while insufficiently modeling business-process dependencies among declaration, transportation, customs clearance, and financial settlement, limiting their ability to construct complete cross-stage evidence chains. Third, current question-answering systems are largely based on generic retrieval-augmented generation and cannot reliably reason over multimodal trade evidence, often producing unsupported timestamps, device records, or risk conclusions. In practical regulatory scenarios, risk explanations must be explicitly supported by documentary fields, sensor records, event timestamps, and confidence estimates rather than textual responses alone [20]. In general, existing research has not yet unified reliability-aware sensor fusion, trade-process-constrained evidence reasoning, and evidence-grounded risk question answering within a single framework.

To address these limitations, TradeSense-EQA, a cross-border trade security anomaly detection framework based on multisource hardware sensing and evidence-grounded English question answering, is proposed. Trade orders, customs declaration batches, or container transportation tasks are treated as the basic analytical units, and trade documents, structured fields, logistics trajectories, hardware sensor sequences, port visual images, and auxiliary financial settlement records are jointly modeled. First, a reliability-aware multisource sensing representation module dynamically estimates modality weights according to missing data, sampling intervals, device health conditions, historical failure rates, and support from neighboring sensors, thereby reducing the influence of equipment noise on risk assessment. The multimodal data are subsequently aligned to a unified trade-process timeline, and physical consistency relationships are examined between declared weight and port weighing records, declared routes and GPS/AIS trajectories, declared quantities and RFID records, and transportation nodes and electronic seal states. On this basis, a trade-process-constrained cross-modal Transformer is employed to locate anomalous events along business nodes and construct a risk evidence chain jointly supported by documentary declarations, physical states, and logistics behaviors. Finally, an evidence-grounded English question-answering module retrieves relevant evidence from the detection results and generates English responses containing risk conclusions, evidence sources, timestamps, and confidence estimates. When the available information is insufficient, an abstention response is produced to reduce factual hallucinations generated by the large language model.

The main contributions of this study are summarized as follows.

1. A multimodal security analysis framework covering the entire cross-border trade process is constructed. Trade documents, structured declaration information, logistics trajectories, RFID, GPS/AIS, electronic seals, weighing records, temperature and humidity measurements, vibration signals, door-state information, and visual data are mapped to a unified trade-process timeline, enabling joint verification of digital declarations and physical facts.
2. A reliability-aware multisource trade sensing representation method is proposed. Information weights are dynamically adjusted according to sensor missingness, noise, drift, and device operating status, thereby improving robustness under equipment failures and incomplete sensing conditions.
3. A trade-process-constrained evidence-chain anomaly detection module is developed. Through process graphs, cross-modal attention, causal masking, and scene-invariant constraints, weight inconsistencies, route deviations, abnormal seal events, cold-chain interruptions, and multistage composite anomalies are identified, and traceable evidence paths are generated for regulatory and auditing applications.
4. An evidence-grounded English risk question-answering module is designed. Fact retrieval, anomaly localization, risk explanation, counterfactual analysis, and financial security association questions are answered on the basis of specific trade documents and sensor records, while unsupported generation is reduced through evidence matching, confidence estimation, and abstention mechanisms.

## 2. Related Work

### 2.1. Cross-Border Trade Security and Anomaly Detection

Cross-border trade anomaly detection aims to identify suspicious transactions by modeling normal trade patterns from declaration information, logistics processes, and financial activities [21]. Early studies mainly relied on expert rules and statistical risk-scoring models based on declared prices, commodity codes, quantities, enterprise profiles, and other structured fields [22]. Although these methods are transparent and easy to deploy, they depend heavily on manually designed rules and cannot effectively capture complex relationships or adapt to evolving fraud strategies [23]. From a financial perspective, such anomalies may further indicate inconsistencies between the declared trade background and the corresponding settlement or transaction activities.

Machine learning and deep learning have substantially improved trade risk detection by learning nonlinear representations from heterogeneous data [24]. Conventional machine learning models, including Random Forest, support vector machine (SVM), XGBoost, and LightGBM, have been widely applied to structured trade data, while pretrained language models analyze trade documents, temporal models capture logistics trajectories, graph neural networks model enterprise and transaction relationships, and financial graph analysis identifies abnormal payment behaviors [25,26,27,28]. These approaches extend trade anomaly detection from structured declarations to semantic, temporal, relational, and financial information.

Despite these advances, most existing methods remain centered on individual data sources [29]. Declaration-based models cannot verify whether declared information is consistent with actual logistics execution, trajectory models provide limited evidence of trade fraud, and financial models lack direct observations of cargo fulfillment [30]. Even multimodal approaches rarely incorporate RFID records, electronic seal states, port weighing data, and environmental sensing as core evidence for trade verification [31]. Consequently, effective cross-border trade security requires unified consistency analysis across documentary declarations, logistics processes, physical sensing, and financial settlement behaviors rather than isolated anomaly detection on individual modalities [32].

### 2.2. Multisource Hardware Sensor Fusion and Logistics Anomaly Perception

Multisource sensor fusion aims to construct a unified representation of logistics states by integrating complementary observations from heterogeneous sensing devices through temporal alignment and cross-modal fusion [33]. In modern cross-border logistics, RFID, GPS, AIS, electronic seals, weighing systems, environmental sensors, X-ray scanners, visual cameras, and edge gateways jointly monitor cargo identity, transportation routes, physical conditions, and device status, providing comprehensive evidence for logistics anomaly perception and trade authenticity verification [34,35].

Existing fusion approaches mainly include early fusion, late fusion, attention mechanisms, and multimodal Transformers [36]. Early and late fusion are limited in capturing cross-modal interactions or are sensitive to missing and noisy observations [37,38]. Attention-based methods dynamically weight multimodal features but do not explicitly model sensor reliability [39], while multimodal Transformers improve cross-modal consistency learning among documentary information, logistics trajectories, and physical sensing data through cross-modal interaction [40].

Standard early fusion combines channel features before their operational quality can be assessed, so a stale gateway packet or a drifted sensor can directly contaminate the shared representation. Standard late fusion is also insufficient in this setting because it combines modality-level decisions without distinguishing a genuine physical event from a confident but unreliable decision produced during intermittent disconnection, packet loss, or sensor drift.

Despite these advances, practical sensing problems—including asynchronous sampling, missed RFID readings, GPS drift, temporary device disconnection, and communication packet loss—remain insufficiently addressed, and sensor reliability is rarely modeled together with trade business processes [41]. Consequently, this study jointly incorporates device health, missing-data patterns, sampling intervals, and neighboring sensor support into reliability estimation, enabling reliable discrimination between equipment failures and genuine logistics anomalies through consistency verification between documentary declarations and hardware-sensed physical evidence.

### 2.3. Evidence-Grounded English Question Answering and Interpretable Risk Analysis

Question-answering systems aim to understand user queries, retrieve relevant information from multimodal sources, and generate semantically consistent responses [42]. Recent advances in pretrained language models, multimodal learning, speech understanding, and retrieval-augmented generation have substantially improved semantic reasoning while reducing factual errors by grounding responses in external knowledge [43,44,45].

Unlike general-purpose question answering, cross-border trade question answering focuses on risk analysis for specific trade orders, customs declarations, or container shipments rather than generic trade knowledge [46]. Answers must be explicitly supported by multimodal evidence, including customs documents, logistics records, sensor observations, trajectories, anomaly detection results, and their corresponding timestamps [47]. When evidence is insufficient, the system should avoid drawing definitive conclusions, particularly for high-risk scenarios such as trade fraud and trade-based money laundering [48].

Existing interpretable risk analysis mainly relies on feature importance, attention visualization, SHAP, and counterfactual explanations [49]. Although these methods explain individual prediction factors, they cannot effectively represent how evidence accumulates across multiple trade stages [50,51]. Evidence-chain reasoning addresses this limitation by organizing abnormal events according to trade processes, providing traceable explanations that better satisfy regulatory auditing requirements [52]. Building upon these advances, this study integrates multimodal anomaly detection, process-oriented evidence chains, and evidence-grounded English question answering to generate trustworthy trade and financial risk analyses with explicit evidence sources, temporal information, and confidence estimates.

## 3. Materials and Method

### 3.1. Data Collection

The cross-border trade security anomaly detection dataset was constructed based on real-world trade processes, where trade document information, logistics transportation records, hardware sensor data, port visual data, speech interaction data, and auxiliary settlement information were jointly associated with the same trade event, as shown in Table 1. Data collection was conducted from January 2023 to December 2025, covering representative international trade hubs including Tianjin Port, Ningbo-Zhoushan Port, and Yantai Port. The collected scenarios involved maritime container transportation, port loading and unloading operations, cold-chain logistics, and cross-border customs clearance processes. Each sample was defined as an individual trade order, customs declaration batch, or container transportation task. Data from heterogeneous sources were aligned through order identifiers, bill-of-lading numbers, container identifiers, enterprise identifiers, and unified timestamps. Trade document data were primarily collected from historical business records provided by cooperating enterprises and publicly available trade information platforms, including commercial contracts, commercial invoices, packing lists, bills of lading, customs declarations, certificates of origin, and inspection and quarantine documents. The collected textual information contained commodity names, HS codes, declared quantities, declared weights, transaction prices, transportation modes, departure ports, destination ports, trading entities, and English commodity descriptions. Logistics trajectory data and hardware sensing data were collected through sensing devices deployed on containers, transportation vehicles, port facilities, and edge gateways. GPS data were continuously acquired from vehicle positioning terminals to record longitude, latitude, velocity, direction, and stop states during transportation. AIS data were obtained from automatic identification system receivers to capture vessel positions, navigation directions, speeds, and maritime transportation trajectories. RFID records were collected through container and cargo identification devices to record the occurrence time of cargo at port gates, storage nodes, and loading or unloading areas. Electronic seal data were obtained from intelligent locking devices, including seal opening and closing states, abnormal opening timestamps, and geographic locations.

Visual data were collected from port surveillance cameras, container inspection devices, and X-ray inspection systems, with acquisition timestamps synchronized with corresponding logistics nodes. The visual dataset mainly consisted of container appearance images, electronic seal region images, cargo loading images, and X-ray inspection images. These visual observations were used to analyze cargo appearance, loading conditions, seal integrity, and consistency between physical cargo states and declared commodity information. English question-answering data were constructed by simulating practical international trade review procedures. Questions were designed by trade compliance specialists, logistics managers, and financial risk assessment personnel according to operational requirements and were recorded in English speech format by professional speakers. The questions covered factual queries, anomaly localization, risk explanation, cross-modal verification, and financial security association analysis. Speech samples were collected using high-quality noise-reduction microphones, while speaking environments, speech rates, and automatic speech recognition (ASR) confidence scores were simultaneously recorded. In addition, auxiliary financial settlement data were collected from trade payment systems and enterprise financial records, including payment timestamps, transaction amounts, currencies, participating entities, and abnormal fund-return relationships.

Across the 13 source categories listed in Table 1, the raw corpus contains 586,180 records, sequences, images, or speech clips. These source-level counts should not be interpreted as 586,180 independent multimodal samples because multiple source items are linked to the same order, declaration batch, or container task. After entity linkage and duplicate removal, the supervised event-level dataset contained 18,420 linked trade events, including 14,238 normal events (77.3%) and 4182 anomalous events (22.7%). The chronological 70%/15%/15% split described in Section 4.1 was performed at this linked-event level. Raw sensor acquisition followed device-specific sampling or event-triggering strategies. GPS trajectories were sampled at 1 Hz, while AIS messages were collected at their native transmission intervals and aggregated to a nominal 10 s interval for event alignment. RFID readers operated in event-triggered inventory windows at approximately 5 Hz when tagged cargo or containers passed a monitored node. Electronic seals and container door-state sensors reported state changes immediately and transmitted heartbeat packets at 60 s intervals. Port weighbridges recorded one stabilized weight measurement for each passage or weighing event. Temperature and humidity measurements were sampled at 0.1 Hz, and vibration signals were sampled at 10 Hz. Port-camera images had a typical native resolution of 1920×1080 pixels, whereas X-ray inspection images were stored at 2048×2048 pixels. All visual inputs were resized to 224×224 pixels for model training, sensor sequences were standardized to 256 time steps, and speech was sampled at 16 kHz.

Ground-truth anomaly labels were not inferred from the model inputs themselves. They were anchored to authoritative post-operation records, including customs physical-inspection outcomes and clearance dispositions, port incident and weighbridge-reconciliation reports, enterprise post-shipment financial reconciliation records and cargo-claim records, and cold-chain quality-control reports. When available, verified electronic-seal incident reports and subsequent inspection records were additionally used to confirm unauthorized opening events. An event was labeled anomalous only when at least one of these authoritative records verified a predefined discrepancy, such as a declaration–weight inconsistency, unauthorized route deviation or seal opening, verified cargo/quantity mismatch, or confirmed cold-chain interruption. Each linked event was independently reviewed by two annotators with complementary expertise: one trade-compliance specialist and one logistics/sensing engineer. The reviewers examined the linked documentary records, operational outcomes, and supporting inspection evidence independently before assigning the event-level anomaly label and anomaly category. Disagreements were adjudicated by a senior trade-risk specialist who was not involved in the initial annotation. Inter-annotator agreement for the binary normal/anomalous decision, measured using Cohen’s κ, was 0.86, indicating strong agreement. All augmented and counterfactual samples inherited labels only when the perturbation preserved the verified event semantics.

The sensing topology comprised three operational layers. At the mobile cargo layer, RFID identifiers were attached to the container or cargo units, the electronic seal and door-state sensor monitored the container doors, and environmental and inertial sensors observed the cargo compartment; vehicle GPS terminals and vessel AIS supplied route information. At the port-fixed layer, RFID readers, weighbridges, surveillance cameras, and X-ray systems were positioned at entry/exit gates, storage nodes, and loading/unloading or inspection areas. At the edge/service layer, gateway records were time-stamped, buffered during temporary outages, associated by container and bill-of-lading identifiers, and forwarded to the event-alignment service. At the field level, UHF RFID communication followed the EPC Class-1 Generation-2 protocol compatible with ISO/IEC 18000-63 [53], while short-range communication between environmental, vibration, door-state, electronic-seal sensors, and local gateways used BLE 5.0 or wired RS-485/Modbus RTU links depending on the deployment location. Mobile gateways used 4G LTE/5G cellular networks as the primary backhaul, whereas fixed port nodes were connected through wired Ethernet or secured Wi-Fi networks. Event and sensor messages were transmitted to the service layer using MQTT over TLS 1.2 with certificate-based device authentication. Locally buffered records were encrypted using AES-256 before synchronization with the central service. This store-and-forward topology permits sealed steel containers to retain locally buffered observations during intermittent connectivity and upload them after the link is restored without treating the outage itself as a cargo anomaly.

### 3.2. Data Augmentation

The fundamental principle of data augmentation is to apply perturbations consistent with real-world operational environments to the original data without altering the core semantics or risk labels of the samples, thereby constructing training instances with different noise levels, missing-data patterns, and presentation forms. The purpose is to expand the training distribution and improve model adaptability to sensor failures, changes in port environments, variations in textual expression, and differences in speech conditions. Let the original multimodal sample be denoted as $X=X^{s},X^{v},X^{t},X^{a}$, where $X^{s}$, $X^{v}$, $X^{t}$, and $X^{a}$ represent sensor time series, visual images, trade text, and English speech, respectively. The augmentation process can be expressed as

(1) $$\tilde{X}=A\left(X;\theta \right),\;y\left(\tilde{X}\right)=y\left(X\right),$$

where A(·) denotes a stochastic augmentation function, θ represents parameters such as perturbation intensity, masking ratio, and transformation probability, and *y* denotes the trade security label of the sample. Label consistency must be preserved throughout augmentation, indicating that the augmented sample should retain the essential risk semantics of the original trade event. For sensor sequences, including GPS, AIS, weight, temperature, humidity, vibration, and device-state signals, random noise injection, temporal masking, drift simulation, and missing-data simulation are applied to reproduce realistic device operating conditions. Given an original time series $x_{t}$, random noise injection is formulated as

(2) $${\tilde{x}}_{t}=x_{t}+\epsilon_{t},\;\epsilon_{t}∼N\left(0,\sigma^{2}\right),$$

where σ controls the noise intensity. This operation is used to simulate weighing errors, environmental fluctuations, and GPS positioning deviations. Long-term sensor drift is represented as

(3) $${\tilde{x}}_{t}=x_{t}+\beta t,$$

where β denotes the drift rate and is used to simulate device aging or calibration bias. To reproduce RFID missed readings, network packet loss, and short-term device disconnection, a binary mask $m_{t}$ is constructed as

(4) $${\tilde{x}}_{t}=m_{t}x_{t}+\left(1-m_{t}\right)x_{\mathrm{miss}},\;m_{t}\in 0,1,$$

where $x_{\mathrm{miss}}$ denotes a missing-value token. If $m_{t}=0$ over a continuous temporal interval, short-term disconnection of electronic seals, edge gateways, or environmental sensors can be simulated. Sampling-interval perturbation is implemented as ${\tilde{t}}_{i}=t_{i}+\delta_{i}$, where $\delta_{i}$ denotes a small random temporal offset. This operation improves robustness to asynchronous sampling and timestamp inaccuracies. Visual augmentation is employed to simulate variations in port camera viewpoints, weather conditions, illumination, and occlusion. Given an original image *I*, geometric transformation is expressed as

(5) $$\tilde{p}=Ap+b,$$

where p and $\tilde{p}$ denote pixel positions before and after transformation, respectively, A controls rotation, scaling, and cropping, and b represents translation. Brightness perturbation is defined as $\tilde{I}=\alpha I+\gamma$, where α and γ control contrast and brightness, respectively. Image blurring is implemented through convolution:

(6) $$\tilde{I}=K*I,$$

where *K* denotes a low-pass or Gaussian convolution kernel. Local occlusion is introduced using a regional mask *M*, such that $\tilde{I}=M⊙I$. Augmented regions are restricted from covering container numbers, electronic seals, cargo contours, and anomalous X-ray regions to prevent the destruction of critical evidence and the introduction of label drift. Text augmentation is designed to account for linguistic variations in contracts, invoices, packing lists, bills of lading, and customs declarations. Let the trade text be represented by the token sequence $T=w_{1},w_{2},\ldots ,w_{n}$. A subset of noncritical tokens can be randomly replaced through masking:

(7) $${\tilde{w}}_{i}=\left\{\begin{array}{cc} {}[\mathrm{MASK}], & r_{i}<p_{m},\\ w_{i}, & r_{i}\geq p_{m}, \end{array}\right.$$

where $p_{m}$ denotes the masking probability. Commodity names, transportation modes, and trade terms can be replaced with semantically equivalent expressions or rewritten in English while preserving their original meanings. The order of noncritical fields can also be adjusted using a permutation function π(·), such that $\tilde{T}=\pi \left(T\right)$. However, fields that determine the risk label, including HS codes, weights, quantities, port names, and container numbers, must not be modified arbitrarily. English speech augmentation is primarily used to simulate port noise, variations in speaking rate, pitch, and accent. Let the original speech signal be denoted as s(t). The augmented speech is formulated as

(8) $$\tilde{s}\left(t\right)=a,s\left(\lambda t\right)+n\left(t\right),$$

where *a* denotes the volume scaling coefficient, λ represents the speaking-rate adjustment coefficient, and n(t) denotes background noise generated by port machinery, vehicles, and crowds. Minor pitch variation can be introduced through the frequency-mapping operator P∗Δf, such that $\tilde{s}=P*\Delta f\left(s\right)$. Accent variation can be simulated by modifying the duration and spectral characteristics of selected phonemes. Nevertheless, the perturbation magnitude must be carefully controlled to avoid altering the actual semantics of the question. In addition to unimodal augmentation, cross-modal counterfactual samples are constructed to enable the contribution of different types of evidence to risk assessment to be learned. Let a sample be represented as $Z=z_{d},z_{r},z_{w},z_{e}$, where $z_{d}$, $z_{r}$, $z_{w}$, and $z_{e}$ correspond to documentary, route, weight, and electronic-seal evidence, respectively. The weight sequence can be replaced while the documentary information remains unchanged, yielding

(9) $$Z_{w}^{cf}=z_{d},z_{r},z_{w}^{cf},z_{e}.$$

The counterfactual contribution of the weight evidence is defined as

(10) $$\Delta_{w}=f\left(Z\right)-f\left(Z_{w}^{cf}\right),$$

where f(·) denotes the risk probability predicted by the model, and $\Delta_{w}$ measures the influence of changes in weight evidence on the risk assessment. Similarly, the electronic seal state can be modified while preserving the transportation trajectory, or RFID node absence can be simulated while retaining the original documentary content. By comparing predictions obtained from original and counterfactual samples, excessive reliance on contextual variables such as ports, countries, or enterprises can be reduced, while relationships between cross-border trade risk and critical evidence, including weight inconsistencies, seal anomalies, route deviations, and cold-chain interruptions, can be learned more accurately.

### 3.3. Proposed Method

#### 3.3.1. Overview

TradeSense-EQA takes standardized, temporally calibrated, and entity-aligned cross-border trade samples as input. Each sample contains trade documents and structured declaration fields, logistics trajectories, multichannel hardware sensor sequences, port visual images, and auxiliary settlement records. Modality-specific encoders are first employed to extract fundamental representations from heterogeneous data. A pretrained language model is used to encode digital declarations concerning commodity descriptions, weights, quantities, routes, and transportation requirements contained in contracts, commercial invoices, packing lists, bills of lading, and customs declarations. A temporal encoding network is used to capture local variations and long-term dependencies in GPS, AIS, RFID, electronic seal, weighing, temperature, humidity, vibration, and container door-state sequences. A visual encoder is used to identify cargo appearance, loading distribution, container numbers, and seal conditions. Structured fields and auxiliary settlement information are mapped into a unified feature space through embedding layers and fully connected networks. The modality-specific representations are subsequently delivered to the reliability-aware multisource trade sensing representation module. Dynamic reliability weights are generated for different sensing channels according to missing-data states, sampling intervals, device health information, historical failure levels, communication quality, and support from neighboring sensors. Asynchronous observations are then mapped to unified trade nodes, including declaration, packing, weighing, port entry, transportation, transshipment, arrival, container opening, customs clearance, and settlement. On this basis, digital declarations contained in trade documents are paired with the corresponding physical facts. For example, declared weights are aligned with port weighing records, planned routes are aligned with GPS or AIS trajectories, and commodity descriptions are aligned with visual cargo characteristics and transportation environments. Unified sensing representations and declaration–fact deviations are thereby obtained for each trade node.

The resulting representations are further delivered to the trade-process-constrained evidence-chain anomaly detection module. Node dependencies are constructed according to the actual business sequence, and information from adjacent stages is propagated through cross-modal attention. Local events, including weight deviations, route departures, seal-opening events, abnormal stops, cold-chain interruptions, and image–text inconsistencies, are jointly evaluated to determine whether they form a continuous composite risk path. Anomaly probabilities, anomaly types, occurrence times, critical devices, node contribution scores, and process-ordered evidence chains are subsequently produced. Finally, the anomaly detection results, original evidence indices, and trade-process node representations are organized into a retrievable risk evidence repository. When an English textual or spoken question is submitted, the speech recognition module first generates the transcribed question and its recognition confidence. The question encoder then identifies the query intent and retrieves relevant evidence from documentary fields, sensor segments, visual regions, anomalous nodes, and auxiliary settlement records. The retrieved evidence and anomaly detection results jointly constrain the large language model during English answer generation. Each response therefore contains a risk judgment, evidence sources, occurrence times, and a confidence estimate. An abstention decision is produced when the available evidence is insufficient or when conflicts exist among different records. Through multitask joint learning, anomaly classification, anomaly localization, evidence selection, answer generation, and confidence prediction are integrated within a unified framework. Consequently, low-level sensing representations can directly support risk identification, while high-level question-answering outputs remain grounded in verifiable trade evidence.

#### 3.3.2. Reliability-Aware Multisource Trade Sensing Representation Module

**Figure 1:**
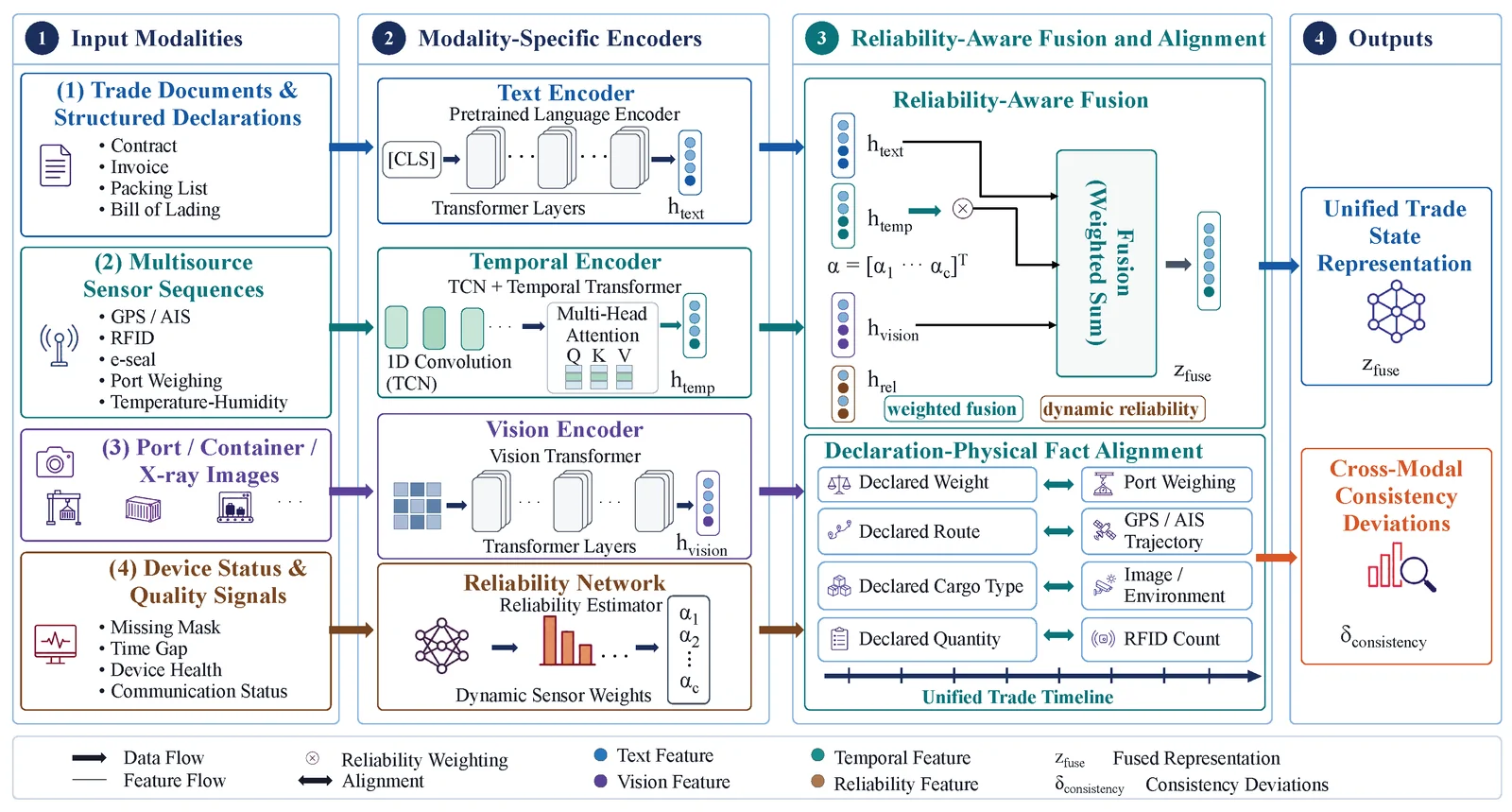
Architecture of the proposed reliability-aware multisource trade sensing representation module. Temporally aligned sensor sequences, port images, and trade documents are first encoded by dedicated temporal, visual, and textual encoders and projected into a shared feature space.

The reliability-aware multisource trade sensing representation module receives temporally calibrated and entity-aligned sensor sequences, port images, and trade document representations, and maps the heterogeneous modalities into a unified *d*-dimensional feature space. As shown in Figure 1, for the *c*-th temporal sensor category, the input window length, variable dimension, and batch size are denoted by $T_{c}$, $F_{c}$, and *B*, respectively. The corresponding input tensor is represented as $X_{c}\in R^{B\times T_{c}\times F_{c}}$. The temporal encoder consists of $L_{c}$ residual temporal convolution blocks and $L_{t}$ Transformer layers. In the *l*-th convolution block, the convolution kernel width, dilation rate, and number of output channels are denoted by $k_{l}$, $\delta_{l}$, and $C_{l}$, respectively. The feature transformation is defined as

(11) $$U_{c}^{\left(l\right)}=\mathrm{LN}\left(U_{c}^{\left(l-1\right)}+P_{l}\left(\sigma \left({Conv1D}_{k_{l},\delta_{l},C_{l}}\left(U_{c}^{\left(l-1\right)}\right)\right)\right)\right),$$

where $P_{l}$ denotes a linear projection used when the input and output channel dimensions are inconsistent. Dilated convolution enables long-distance transportation patterns to be covered without substantially increasing the number of model parameters. The temporal features are subsequently passed through a multi-head self-attention layer:

(12) $$S_{c}^{\left(j\right)}=\mathrm{LN}\left(S_{c}^{\left(j-1\right)}+{\mathrm{MHA}}_{h_{j},d_{j}}\left(S_{c}^{\left(j-1\right)}\right)\right),$$

where $h_{j}$ and $d_{j}$ denote the number of attention heads and the hidden width of the *j*-th layer, respectively. For a port image with spatial dimensions $H_{v}\times W_{v}$ and $C_{v}$ channels, image patches of size $H_{p}\times W_{p}$ are generated, resulting in $N_{v}=H_{v}W_{v}/\left(H_{p}W_{p}\right)$ visual tokens. These tokens are processed by an $L_{v}$-layer Vision Transformer to extract container numbers, seal conditions, and cargo distribution characteristics. After being encoded by an $L_{d}$-layer text encoder, the document semantic representation is projected together with the temporal and visual representations into the shared *d*-dimensional space.

To prevent device failures from being misclassified as trade anomalies, a reliability vector is constructed for each sensor by jointly considering the missing-data mask $m_{c}$, sampling interval $\tau_{c}$, device health state $g_{c}$, communication quality $q_{c}$, and support from neighboring devices $a_{c}$:

(13) $$r_{c}=\mathrm{sigmoid}\left(W_{r}\left[m_{c}∥\phi \left(\tau_{c}\right)∥g_{c}∥q_{c}∥a_{c}\right]+b_{r}\right).$$

All reliability inputs are calculated over the same aligned window and normalized to [0, 1]. Specifically, $m_{c}$ contains the observed-sample ratio and the longest-missing-run ratio; $\phi (\tau_{c})$ contains the normalized deviation of the observed inter-arrival interval from the nominal sampling interval and its coefficient of variation. Device health is defined as $g_{c}=\left[b_{c},1-f_{c},\exp\left(-d_{c}^{\mathrm{cal}}/T_{\mathrm{cal}}\right),1-h_{c}\right]$, where $b_{c}$ is normalized battery level, $f_{c}$ is the self-diagnostic fault flag, $d_{c}^{\mathrm{cal}}$ is time since calibration, and $h_{c}$ is the historical failure rate. Communication quality is $q_{c}=\left[p_{c}^{\mathrm{del}},{\tilde{\mathrm{RSSI}}}_{c},\exp\left(-\ell_{c}/T_{q}\right)\right]$, where $p_{c}^{\mathrm{del}}$ is packet-delivery ratio, ${\tilde{\mathrm{RSSI}}}_{c}$ is normalized received-signal strength, and $\ell_{c}$ is median gateway latency. Neighboring-device support is computed as

$$a_{c}=\frac{\sum_{u\in N_{c}}\eta_{cu}\exp\;\left(-{∥P_{c}S_{c}-P_{u}S_{u}∥}_{2}/\kappa \right)}{\sum_{u\in N_{c}}\eta_{cu}+\epsilon },$$

where $N_{c}$ contains co-located or process-adjacent devices observing the same event, $P_{c}$ maps channel *c* to the shared space, $\eta_{cu}$ is zero for temporally invalid neighbors and otherwise reflects temporal overlap, κ controls agreement sensitivity, and ϵ prevents division by zero. Device and gateway logs supply $g_{c}$ and $q_{c}$, while $a_{c}$ is calculated from aligned observations rather than manually assigned.

To obtain consistent reliability-aware weights across heterogeneous modalities, the visual and document representations are additionally associated with modality-specific reliability logits $r_{v}$ and $r_{d}$. Specifically, $r_{v}$ is estimated from visual availability and quality indicators, such as missing-image status and image-quality information, whereas $r_{d}$ is estimated from document availability, OCR confidence, and field completeness. Let M={1,…,C,v,d} denote the complete set of projected temporal sensor, visual, and document modalities. A unified normalized gating mechanism is then applied to all modalities:

(14) $$\alpha_{i}=\frac{\exp(r_{i}/\gamma )}{\sum_{j\in M}\exp\left(r_{j}/\gamma \right)},\;i\in M,$$

and the fused representation is calculated as

(15) $$Z=\sum_{c=1}^{C}\alpha_{c}W_{c}S_{c}+\alpha_{v}W_{v}V+\alpha_{d}W_{d}D.$$

Here, γ controls the smoothness of the weight distribution. Consequently, unreliable sensing channels can be down-weighted while reliable visual and documentary evidence can receive correspondingly larger contributions. Declaration–fact consistency is measured through a projected similarity function:

(16) $$e_{pq}=1-\frac{{\left(W_{p}z_{p}\right)}^{⊤}\left(W_{q}z_{q}\right)}{{\left|W_{p}z_{p}\right|}_{2}{\left|W_{q}z_{q}\right|}_{2}},$$

where $z_{p}$ denotes a digital declaration, such as a declared weight, planned route, or commodity description, and $z_{q}$ denotes a physical fact derived from weighing, trajectory, environmental, or visual sensing data. The weighted fusion mechanism has a conditional robustness property rather than an unconditional guarantee. To include the visual and document terms in Equation (14), let M={1,…,C,v,d} index all projected modalities, let $Z=\sum_{i\in M}\alpha_{i}{\hat{z}}_{i}$, and define $\varepsilon_{i}={\hat{z}}_{i}-z^{*}$, with $\alpha_{i}\geq 0$ and $\sum_{i}\alpha_{i}=1$. The triangle inequality gives

(17) $${∥Z-z^{*}∥}_{2}\leq B\left(\alpha \right)=\sum_{i\in M}\alpha_{i}{∥\varepsilon_{i}∥}_{2}.$$

Suppose only the reliability logit of a faulty channel *c* is reduced while the other logits remain fixed. Under the normalized softmax gate, $\delta_{i}=\alpha_{i}^{\prime }-\alpha_{i}\geq 0$ for i≠c, $\delta_{c}<0$, and $\sum_{i\neq c}\delta_{i}=-\delta_{c}=\alpha_{c}-\alpha_{c}^{\prime }$. The exact change in the error upper bound is therefore

(18) $$B\left(\alpha^{\prime }\right)-B\left(\alpha \right)=\left(\alpha_{c}-\alpha_{c}^{\prime }\right)\left({\bar{e}}_{-c}^{\;\delta }-{∥\varepsilon_{c}∥}_{2}\right),\;{\bar{e}}_{-c}^{\;\delta }=\frac{\sum_{i\neq c}\delta_{i}{∥\varepsilon_{i}∥}_{2}}{\alpha_{c}-\alpha_{c}^{\prime }}.$$

Consequently, down-weighting channel *c* tightens this bound only under the additional assumption ${∥\varepsilon_{c}∥}_{2}>{\bar{e}}_{-c}^{\;\delta }$; that is, the faulty channel must have greater error than the redistribution-weighted average error of the receiving sensor, visual, and document modalities.

#### 3.3.3. Trade-Process-Constrained Evidence-Chain Anomaly Detection Module

**Figure 2:**
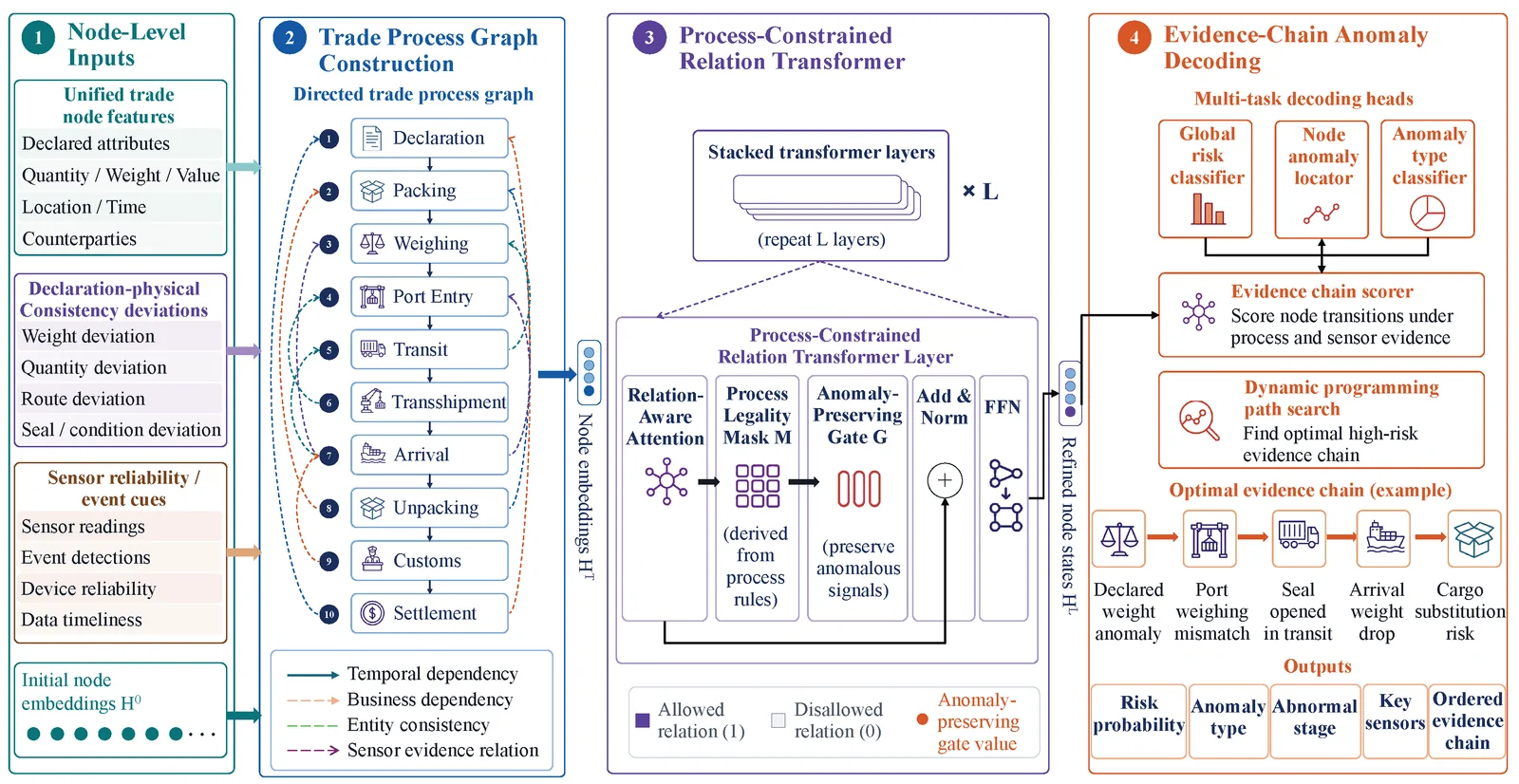
Architecture of the proposed trade-process-constrained evidence-chain anomaly detection module. Trade-state representations and declaration–fact consistency deviations are first organized into a directed trade-process graph, where nodes represent trade stages and edges encode temporal, business, and evidence relationships.

The trade-process-constrained evidence-chain anomaly detection module receives the trade-node representations and declaration–fact deviations produced by the preceding module and organizes declaration, packing, weighing, port entry, transportation, transshipment, arrival, container opening, customs clearance, and settlement into a directed process graph. As shown in Figure 2, the batch size, number of process nodes, and node-feature width are denoted by *B*, *N*, and $d_{g}$, respectively. The input tensor is represented as $H^{\left(0\right)}\in R^{B\times N\times d_{g}}$. Multitype evidence relationships among nodes are represented by $R\in R^{B\times N\times N\times C_{e}}$, where $C_{e}$ denotes the number of relation channels describing temporal dependencies, business dependencies, weight consistency, trajectory consistency, and device events.

The module consists of $L_{g}$ process-constrained Transformer layers. The *l*-th layer contains $h_{l}$ attention heads, and the query, key, and value widths of each head are denoted by $d_{l}^{q}$, $d_{l}^{k}$, and $d_{l}^{v}$, respectively. The intermediate width of the feed-forward network is denoted by $d_{l}^{f}$. For the *a*-th attention head, the relation-aware score from node *i* to node *j* is defined as

(19) $$s_{ij}^{\left(l,a\right)}=\frac{\left(H_{i}^{\left(l-1\right)}W_{Q}^{\left(l,a\right)}\right){\left(H_{j}^{\left(l-1\right)}W_{K}^{\left(l,a\right)}\right)}^{⊤}}{\sqrt{d_{l}^{k}}}+\psi_{l,a}\left(R_{ij}\right)+\omega_{l,a}\Delta t_{ij}+M_{ij},$$

where $\psi_{l,a}\left(\cdot \right)$ denotes a relation-channel mapping function, $\Delta t_{ij}$ represents the temporal interval between two trade nodes, and $M_{ij}$ denotes the process-constraint mask. When node *j* is not a valid predecessor, successor, or authorized associated node of node *i*, $M_{ij}$ is set to −∞; otherwise, it is set to 0. The normalized attention weight and node update are given by

(20) $$\beta_{ij}^{\left(l,a\right)}=\frac{\exp\left(s_{ij}^{\left(l,a\right)}\right)}{\sum_{u=1}^{N}\exp\left(s_{iu}^{\left(l,a\right)}\right)},\;{\tilde{H}}_{i}^{\left(l\right)}=\overset{h_{l}}{\underset{a=1}{∥}}\sum_{j=1}^{N}\beta_{ij}^{\left(l,a\right)}H_{j}^{\left(l-1\right)}W_{V}^{\left(l,a\right)},$$

and

(21) $$H^{\left(l\right)}=\mathrm{LN}\left({\tilde{H}}^{\left(l\right)}W_{O}^{\left(l\right)}+H^{\left(l-1\right)}\right)+{\mathrm{FFN}}_{d_{l}^{f}}\left(\mathrm{LN}\left({\tilde{H}}^{\left(l\right)}W_{O}^{\left(l\right)}+H^{\left(l-1\right)}\right)\right).$$

After propagation through $L_{g}$ layers, a binary classification head, a multilabel anomaly head, and a node-localization head are used to estimate the overall risk probability, anomaly-category probabilities, and node-level anomaly scores:

(22) $$p^{\mathrm{risk}}=\sigma \left(W_{b}\mathrm{Pool}\left(H^{\left(L_{g}\right)}\right)+b_{b}\right),\;p_{i}^{\mathrm{node}}=\sigma \left(W_{n}H_{i}^{\left(L_{g}\right)}+b_{n}\right).$$

The evidence chain is not constructed by simply selecting several nodes with the highest anomaly scores. Instead, an optimal path is searched in the process graph by jointly considering node abnormality, evidence consistency, and path continuity. For a candidate path $\pi =(v_{1},\ldots ,v_{K})$, its score is defined as

(23) $$S\left(\pi \right)=\sum_{k=1}^{K}\log p_{v_{k}}^{\mathrm{node}}+\lambda_{r}\sum_{k=1}^{K-1}\chi \left(R_{v_{k}v_{k+1}}\right)-\lambda_{t}\sum_{k=1}^{K-1}\rho \left(\Delta t_{v_{k}v_{k+1}}\right).$$

The optimal evidence chain is obtained through dynamic programming:

(24) $$\pi^{*}=\underset{\pi \in P}{\arg\max},S\left(\pi \right),$$

where P contains only valid paths that satisfy both business-order and temporal-order constraints. The process constraint has an explicit mathematical guarantee. If no valid process relationship exists between nodes *i* and *j*, then $M_{ij}=-\infty$, and therefore

(25) $$\beta_{ij}^{\left(l,a\right)}=\frac{\exp(-\infty )}{\sum_{u=1}^{N}\exp\left(s_{iu}^{\left(l,a\right)}\right)}.$$

Consequently, invalid nodes cannot propagate information to the current node through the attention mechanism. Model predictions therefore depend only on node sets permitted by the actual business process, preventing temporally reversed events, unrelated ports, or nonadjacent trade stages from being combined into spurious evidence chains. Since $\pi^{*}$ is restricted to the valid path set P, the resulting evidence chain inherently satisfies process continuity. This design enables dispersed events, such as declared-weight deviations, abnormal port weighing records, seal-opening events during transportation, and reduced arrival weights, to be connected into a complete risk path. The model therefore not only produces anomaly labels but also localizes abnormal stages, identifies critical devices, and supplies temporally ordered and business-grounded evidence for subsequent English risk question answering.

#### 3.3.4. Evidence-Grounded English Risk Question-Answering Module

**Figure 3:**
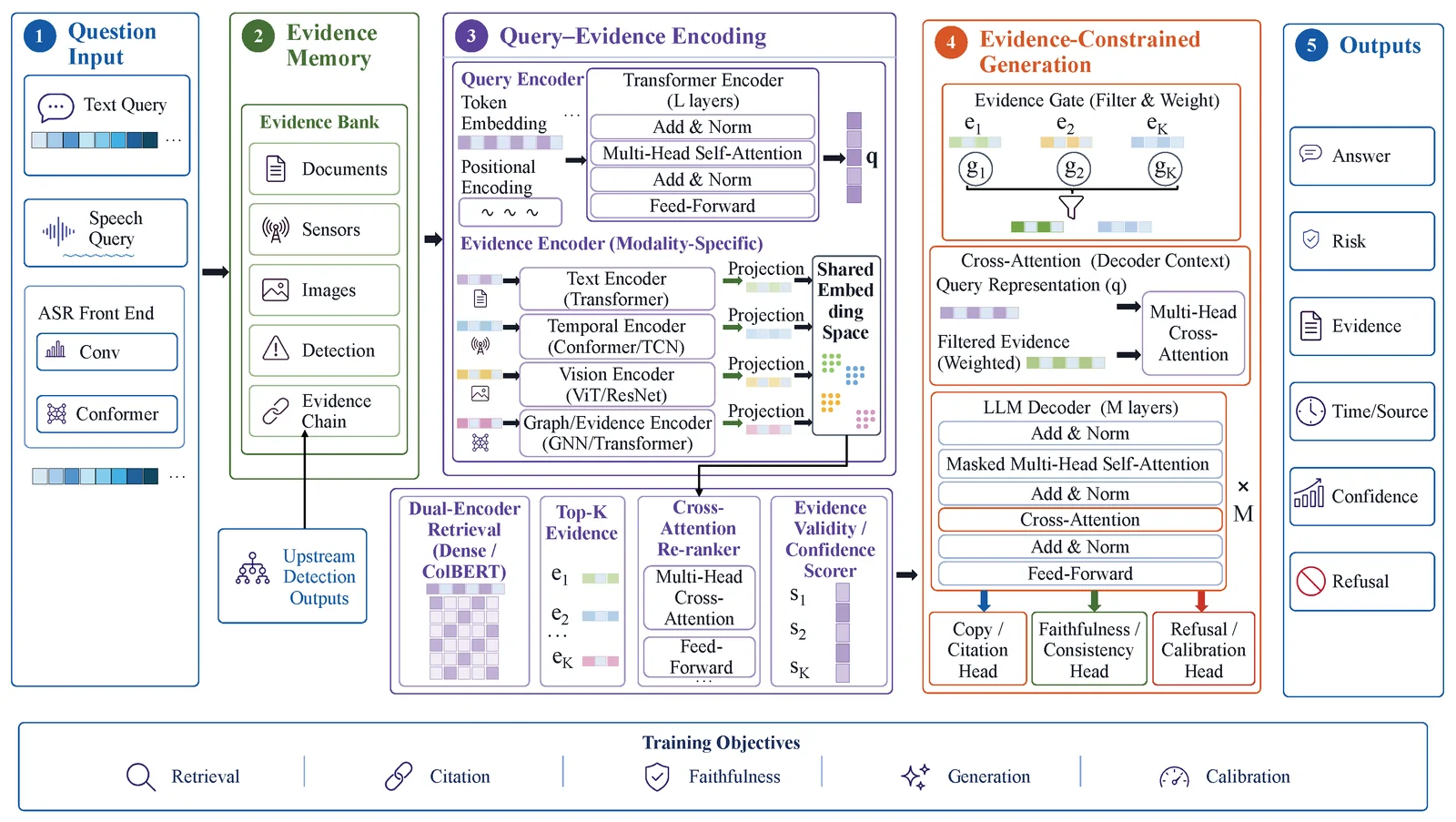
Architecture of the proposed evidence-grounded English risk question-answering module. Spoken or typed English questions are first encoded into semantic representations and matched against a multimodal evidence repository containing trade documents, sensor records, anomaly nodes, and evidence chains.

The evidence-grounded English risk question-answering module receives node representations, anomaly categories, node-level risk scores, the optimal evidence chain, and original document and sensor indices produced by the trade-process-constrained anomaly detection module. These elements are organized into a retrievable multimodal evidence repository. As shown in Figure 3, for a spoken question, the acoustic feature tensor is denoted by $A\in R^{B\times T_{a}\times F_{a}}$, where $T_{a}$ and $F_{a}$ represent the number of speech frames and the acoustic feature width, respectively. The acoustic front end consists of an $L_{a}$-layer convolutional subsampling network and an $L_{c}$-layer Conformer encoder. Their kernel size, output channel number, and hidden width are denoted by $K_{a}$, $C_{a}$, and $d_{a}$, respectively. After the speech representation has been extracted, a connectionist temporal classification decoder is used to generate the English transcription and the corresponding ASR confidence score.

For a keyboard-entered question, the token sequence $Q\in R^{B\times L_{q}}$ is directly passed into a question encoder composed of $L_{q}^{e}$ Transformer layers. Each layer contains $H_{q}$ attention heads, with hidden and feed-forward widths denoted by $d_{q}$ and $d_{q}^{f}$, respectively. The resulting question representation is denoted by $z_{q}$. Heterogeneous trade evidence is represented as $E\in R^{B\times N_{e}\times d_{e}}$. Documentary fields, temporal segments, visual regions, anomalous nodes, and settlement records are separately projected into a common width $d_{e}$. When visual evidence is derived from a feature map with dimensions $H_{v}\times W_{v}$ and $C_{v}$ channels, regional pooling is applied to obtain localized evidence tokens. Question–evidence relevance is first estimated using a dual-encoder retriever:

(26) $$\gamma_{j}=\frac{\exp\left({\left(P_{q}z_{q}\right)}^{⊤}\left(P_{e}E_{j}\right)/\tau_{q}\right)}{\sum_{u=1}^{N_{e}}\exp\left({\left(P_{q}z_{q}\right)}^{⊤}\left(P_{e}E_{u}\right)/\tau_{q}\right)},$$

where $\tau_{q}$ denotes a temperature parameter. The top-$K_{e}$ evidence candidates are subsequently passed to an evidence reranker consisting of $L_{r}$ cross-attention layers. Evidence source, timestamp, device reliability, node-level risk score, and process position are jointly incorporated to estimate an evidence-validity score:

(27) $$\xi_{j}=w_{\xi }^{⊤}\mathrm{GELU}\left(W_{\xi }\left[z_{q}∥E_{j}∥t_{j}∥s_{j}∥r_{j}\right]+b_{\xi }\right).$$

To prevent the language model from accessing irrelevant or insufficiently reliable information, an evidence-permission mask $G_{j}$ is defined. Only evidence that simultaneously satisfies retrieval relevance, device reliability, and process-consistency requirements is made visible:

(28) $$G_{j}=\left\{\begin{array}{cc} 0, & \gamma_{j}\geq \theta_{\gamma },\;\xi_{j}\geq \theta_{\xi },\;r_{j}\geq \theta_{r},\\ -\infty , & \mathrm{otherwise}. \end{array}\right..$$

A question-specific context is then obtained through evidence-constrained cross-attention:

(29) $$C_{q}=\mathrm{softmax}\left(\frac{\left(z_{q}W_{Q}\right){\left(EW_{K}\right)}^{⊤}}{\sqrt{d_{k}}}+G\right)EW_{V}.$$

The context $C_{q}$, question representation, anomaly category, and evidence-chain encoding are subsequently passed to a large-language-model decoder containing $L_{g}$ layers, with hidden width $d_{g}$, $H_{g}$ attention heads, and feed-forward width $d_{g}^{f}$. To enable direct citation of weight values, port names, device identifiers, and timestamps, the output layer combines vocabulary generation with evidence copying:

(30) $$P\left(y_{t}\right)=\lambda_{t}P_{\mathrm{vocab}}\left(y_{t}\right)+\left(1-\lambda_{t}\right)\sum_{j:E_{j}=y_{t}}P_{\mathrm{copy}}\left(E_{j}\right),$$

where

(31) $$\lambda_{t}=\sigma \left(w_{\lambda }^{⊤}\left[h_{t}∥C_{q}∥e\left(y_{t-1}\right)\right]+b_{\lambda }\right).$$

An evidence-citation head, an answer-consistency head, and an abstention head are jointly incorporated. Answer confidence is estimated from the generation probability, evidence coverage, ASR confidence, and inter-evidence consistency:

(32) $$c_{\mathrm{ans}}=\varphi \left(\frac{1}{T_{y}}\sum_{t=1}^{T_{y}}\log P\left(y_{t}\right),\sum_{j}\gamma_{j}I\left(G_{j}=0\right),c_{\mathrm{asr}},c_{\mathrm{evi}}\right).$$

When $c_{\mathrm{ans}}<\theta_{c}$ or the number of valid evidence items falls below a predefined threshold, an abstention response indicating insufficient evidence is produced. The joint training objective consists of answer-generation, evidence-retrieval, citation-localization, factual-consistency, and abstention-calibration losses:

(33) $$L_{\mathrm{EQA}}=\lambda_{\mathrm{gen}}L_{\mathrm{gen}}+\lambda_{\mathrm{ret}}L_{\mathrm{ret}}+\lambda_{\mathrm{cite}}L_{\mathrm{cite}}+\lambda_{\mathrm{faith}}L_{\mathrm{faith}}+\lambda_{\mathrm{ref}}L_{\mathrm{ref}}.$$

The five coefficients are fixed training hyperparameters, not dynamically estimated fusion weights; the distinct λ notation avoids conflict with the sensor weights $\alpha_{c}$. Their values were $\lambda_{\mathrm{gen}}=1.0$, $\lambda_{\mathrm{ret}}=0.5$, $\lambda_{\mathrm{cite}}=0.3$, $\lambda_{\mathrm{faith}}=0.5$, and $\lambda_{\mathrm{ref}}=0.2$. They were selected using a coarse-to-fine coordinate grid search on the validation set, with $\lambda_{\mathrm{ret}}$, $\lambda_{\mathrm{cite}}$, and $\lambda_{\mathrm{faith}}$ searched over {0.25,0.5,0.75,1.0} and $\lambda_{\mathrm{ref}}$ searched over {0.1,0.2,0.3,0.5}. The same validation-only procedure selected γ, $K_{e}$, $\theta_{\gamma }$, $\theta_{\xi }$, $\theta_{r}$, $\theta_{c}$, and the minimum valid-evidence count; their deployed values are γ=0.7, $K_{e}=10$, $\theta_{\gamma }=0.05$, $\theta_{\xi }=0.50$, $\theta_{r}=0.60$, $\theta_{c}=0.65$. The proposed constraint mechanism provides an explicit evidence-exclusion property. For any evidence item $E_{j}$ that does not satisfy the permission conditions, $G_{j}=-\infty$. Its cross-attention weight therefore satisfies

(34) $$\lim_{G_{j}\to -\infty }\frac{\exp\left(u_{j}+G_{j}\right)}{\sum_{m=1}^{N_{e}}\exp\left(u_{m}+G_{m}\right)}.$$

Thus, irrelevant, unreliable, or process-inconsistent evidence cannot enter the generation context, and its direct contribution to the answer is strictly eliminated. If the valid evidence set is denoted by $E^{+}$, the generation context can be represented as a weighted combination over $E^{+}$. Consequently, verifiable facts contained in the generated answer originate only from permitted documentary fields, sensor records, and risk nodes. This design reduces the generation of fabricated weights, timestamps, and device events by the large language model. It also supports factual queries, anomaly localization, risk explanation, cross-modal verification, counterfactual analysis, and financial-security association questions, while ensuring linguistic readability, evidence traceability, and practical auditability.

## 4. Results and Discussion

### 4.1. Experimental Configuration

#### 4.1.1. Hardware and Software Platform

All experiments were conducted on a workstation equipped with an Intel Xeon Gold 6330 processor, 256 GB of DDR4 memory, and two NVIDIA RTX 4090 GPUs, each providing 24 GB of video memory. A 4 TB NVMe solid-state drive was used for data storage to support the high-throughput loading of trade documents, sensor time series, port images, and English speech data. The GPUs were primarily used to train deep learning models, including Transformer, BERT, CLIP, VisualBERT, and BERT-QA, whereas the CPU was used for data cleaning, entity alignment, feature preprocessing, and the training of conventional machine learning models. All experiments were performed on the same hardware platform to ensure the comparability of training efficiency and detection performance across different methods. Ubuntu 22.04 LTS was adopted as the operating system, and Python 3.10 was used as the programming environment. PyTorch 2.2 served as the primary deep learning framework, with CUDA 12.1 and cuDNN 8.9 configured for GPU acceleration. Textual models were implemented using Transformers 4.38. OpenCV 4.9 and Torchvision 0.17 were used for image processing, while Librosa 0.10 and Torchaudio 2.2 were employed for speech preprocessing and feature extraction. Conventional machine learning models were implemented using Scikit-learn 1.4, XGBoost 2.0, and LightGBM 4.3. Data processing was performed with Pandas 2.2 and NumPy 1.26, and graph structures and evidence chains were modeled using PyTorch Geometric 2.5. Statistical analysis and result visualization were conducted using SciPy (v1.13.1), Matplotlib (v3.9.0), and Seaborn (v0.13.2).

The dataset was chronologically divided into training, validation, and test subsets according to the occurrence times of trade events, with proportions of 70%, 15%, and 15%, respectively. Associated records belonging to the same enterprise, order, bill of lading, or container were prevented from appearing in more than one subset. Five-fold cross-validation was performed within the training set for hyperparameter selection. In each fold, four subsets were used for training and the remaining subset was used for validation. The mean and standard deviation obtained over the five folds were subsequently reported. The models were optimized using AdamW. The initial learning rate was set to $1\times 10^{-4}$, while the learning rate for the pretrained textual and visual encoders was set to $2\times 10^{-5}$. The weight-decay coefficient was set to $1\times 10^{-4}$, the batch size was set to 32, and the maximum number of training epochs was set to 100. Early stopping was applied when validation performance failed to improve for 10 consecutive epochs. Linear learning-rate warm-up was performed during the first 5% of the training steps, followed by cosine annealing. The gradient-clipping threshold was set to 1.0, and the dropout rate was set to 0.2. The hidden dimension of the Transformer was set to 256, with 8 attention heads, 4 encoding layers, and a feed-forward dimension of 1024. The TCN kernel size was set to 3, and the dilation rates were sequentially configured as 1, 2, 4, and 8. The maximum text length was limited to 512 tokens, sensor sequences were standardized to 256 time steps, input images were resized to 224×224, and the speech sampling rate was set to 16 kHz. In the multitask objective, the weights assigned to anomaly classification, event localization, evidence matching, and English question answering were set to 1.0, 0.5, 0.5, and 1.0, respectively. A fixed random seed of 42 was used, and each experiment was independently repeated five times.

#### 4.1.2. Baseline Models and Evaluation Metrics

The baseline models included XGBoost [54], LightGBM [55], TCN [56], Transformer [57], BERT [58], CLIP [59], VisualBERT [60], BERT-QA [58], MulT [61], and Perceiver IO [62]. XGBoost is an ensemble learning model based on gradient-boosted decision trees, in which classification is achieved by iteratively fitting the residuals of preceding learners. Its main advantage lies in its strong nonlinear modeling capability for structured trade data. LightGBM adopts a leaf-wise gradient-boosting strategy and a histogram-based algorithm, providing high training efficiency and relatively low memory consumption.

TCN employs dilated causal convolutions to extract local and long-range characteristics from sensor sequences. Its advantages include parallelizable training and preservation of temporal causality. Transformer estimates dependencies among different sequence positions through self-attention and is therefore well suited to modeling long-range logistics processes and sensor-state variations. BERT applies a bidirectional Transformer to encode trade document text, allowing contextual information to be fully exploited for detecting contradictory fields and semantic anomalies. CLIP aligns visual and textual representations through contrastive learning and is therefore suitable for evaluating consistency between cargo images and declared descriptions. VisualBERT jointly encodes visual-region features and textual tokens, enabling fine-grained interactions between images and documents to be learned. BERT-QA predicts the start and end positions of an answer span based on BERT representations and can therefore extract answers from trade documents and evidence text with high precision.

To provide stronger multimodal comparisons under matched input conditions, MulT and Perceiver IO were additionally included. MulT employs directional pairwise cross-modal attention to capture interactions among heterogeneous modality sequences without requiring explicit temporal alignment, making it suitable for jointly modeling asynchronous documentary, trajectory, sensing, and visual information. Perceiver IO maps heterogeneous and potentially high-dimensional inputs into a compact latent representation through cross-attention and provides a general architecture for integrating structured inputs from different data domains. In our implementation, both MulT and Perceiver IO were supplied with the same complete set of input modalities as TradeSense-EQA, including structured declaration fields, trade-document text, GPS/AIS trajectories, multichannel hardware-sensor sequences, port and container images, and auxiliary settlement records. For MulT, modality-specific encoders first transformed these sources into token or temporal sequences, which were subsequently fused through cross-modal attention. For Perceiver IO, modality-specific embeddings together with positional and modality identifiers were mapped into the shared latent array before anomaly classification.

The modality access of all comparison methods was therefore explicitly controlled and documented as follows. XGBoost and LightGBM received structured declaration and auxiliary settlement features; TCN and Transformer received aligned trajectories and hardware-sensor sequences; BERT and BERT-QA received trade-document text; and CLIP and VisualBERT received paired document text and port images. In contrast, MulT, Perceiver IO, and TradeSense-EQA all received the same complete multimodal input set consisting of structured fields, trade documents, trajectories, hardware-sensor sequences, images, and auxiliary settlement records. Accordingly, the comparisons with XGBoost, LightGBM, TCN, Transformer, BERT, CLIP, VisualBERT, and BERT-QA evaluate complete deployable systems under their conventional modality assumptions, whereas the comparisons with MulT and Perceiver IO provide same-input multimodal controls for more directly assessing architectural effectiveness.

Accuracy, Precision, Recall, Macro-F1, ROC-AUC, Event Localization Precision, Event Localization Recall, Evidence F1, Evidence Hit@*K*, Counterfactual Validity, Exact Match, Token-level F1, BLEU, ROUGE-L, and BERTScore were adopted as evaluation metrics.

(35) $$\begin{array}{c} Acc=\frac{TP+TN}{TP+TN+FP+FN},\;P=\frac{TP}{TP+FP},\;R=\frac{TP}{TP+FN},\\ F1=\frac{2PR}{P+R},\;Macro\text{-}F1=\frac{1}{C}\sum_{c=1}^{C}F1_{c}. \end{array}$$

(36) $$AUC=P\left(s^{+}>s^{-}\right),\;P_{loc}=\frac{N_{loc}^{TP}}{N_{loc}^{TP}+N_{loc}^{FP}},\;R_{loc}=\frac{N_{loc}^{TP}}{N_{loc}^{TP}+N_{loc}^{FN}},$$

(37) $$F1_{e}=\frac{2P_{e}R_{e}}{P_{e}+R_{e}},\;Hit@K=\frac{1}{N}\sum_{i=1}^{N}I\left(E_{i}\cap {\hat{E}}_{i}^{K}\neq ⌀\right),\;CV=\frac{1}{N}\sum_{i=1}^{N}I\left[f\left(x_{i}\right)\neq f\left(x_{i}^{cf}\right)\right],$$

(38) $$EM=\frac{1}{N}\sum_{i=1}^{N}I\left({\hat{y}}_{i}=y_{i}\right),\;F1_{tok}=\frac{2P_{tok}R_{tok}}{P_{tok}+R_{tok}},\;BLEU=BP\exp\left(\sum_{n=1}^{M}w_{n}\ln p_{n}\right),$$

(39) $$ROUGE\text{-}L=\frac{\left(1+\beta^{2}\right)P_{LCS}R_{LCS}}{R_{LCS}+\beta^{2}P_{LCS}},\;BERTScore=\frac{2P_{B}R_{B}}{P_{B}+R_{B}}.$$

Here, TP, TN, FP, and FN denote the numbers of true positives, true negatives, false positives, and false negatives, respectively. *C* denotes the number of classes, while $s^{+}$ and $s^{-}$ represent the prediction scores assigned to positive and negative samples. $N_{loc}$ denotes the number of localized anomalous events. $E_{i}$ and ${\hat{E}}_{i}^{K}$ denote the ground-truth evidence set and the top-*K* predicted evidence items, respectively, and $x_{i}^{cf}$ denotes a counterfactual sample. BP denotes the brevity penalty, $p_{n}$ represents the modified precision of *n*-grams, LCS denotes the longest common subsequence, and $P_{B}$ and $R_{B}$ denote semantic similarity-based precision and recall, respectively.

### 4.2. Cross-Border Trade Security Anomaly Detection Task

This experiment was designed to compare the detection capabilities of different model categories in the cross-border trade security anomaly detection task. Particular emphasis was placed on evaluating the respective contributions of structured declaration information, sensor time series, trade documents, and visual evidence to anomaly identification, as well as on assessing the overall advantage of TradeSense-EQA under multisource heterogeneous information fusion.

**Figure 4:**
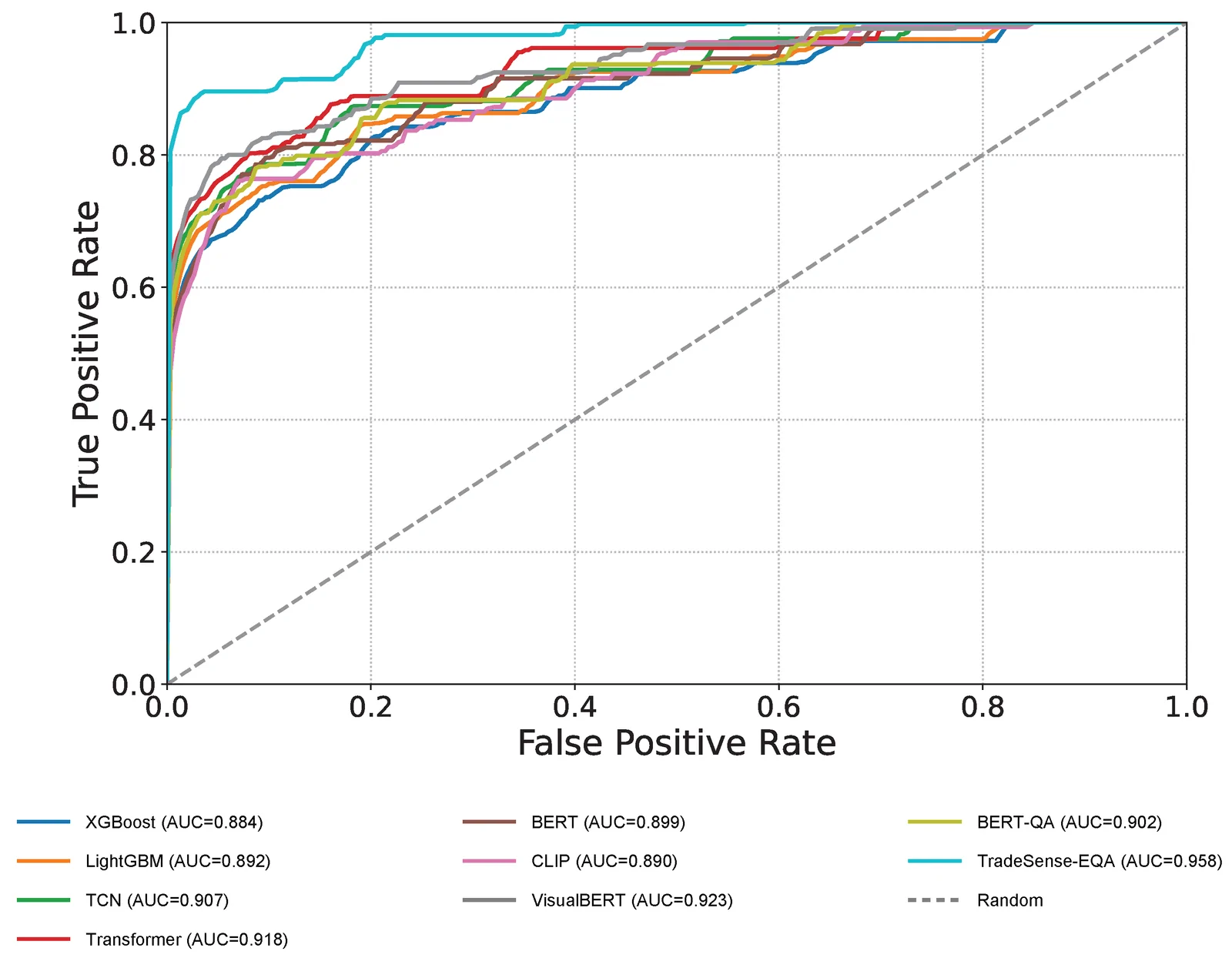
ROC curve comparison of different methods on the cross-border trade security anomaly detection task. The ROC-AUC values in the legend report the five-run means and have been reconciled with Table 2; TradeSense-EQA demonstrates the strongest discrimination between normal and anomalous trade samples.

As shown in Table 2 and Figure 4, XGBoost achieved an Accuracy of 0.842 and a Macro-F1 of 0.817, indicating that gradient-boosted decision trees can effectively learn nonlinear combinations among declared prices, weights, quantities, and enterprise attributes. However, its Recall was only 0.806, suggesting that complex anomalies dependent on continuous logistics processes were still frequently missed. LightGBM improved the Accuracy, Macro-F1, and ROC-AUC to 0.851, 0.828, and 0.892, respectively, demonstrating that its leaf-wise growth strategy enabled a more refined partitioning of high-risk samples. TCN obtained a Macro-F1 of 0.846 and a ROC-AUC of 0.907, indicating that dilated causal convolutions were effective in extracting both local and long-range patterns from sensor sequences, including seal-state changes, weight fluctuations, and environmental anomalies. Transformer further increased the ROC-AUC to 0.918, reflecting the advantage of self-attention in modeling long-range dependencies across trade-process nodes. BERT achieved a Macro-F1 of 0.836, confirming that contextual semantics in trade documents can support the identification of contradictory fields, although textual information alone cannot verify physical fulfillment conditions. The overall performance of CLIP remained relatively limited, suggesting that global image–text alignment is insufficient for resolving fine-grained trade anomalies. VisualBERT increased the Macro-F1 to 0.866 by jointly encoding visual regions and textual tokens. BERT-QA outperformed BERT, but its primary emphasis on answer-span extraction limited its ability to model anomalies distributed across the entire trade process.

TradeSense-EQA achieved the best performance across all evaluation metrics, with an Accuracy of 0.918, Recall of 0.897, Macro-F1 of 0.903, and ROC-AUC of 0.958. Its comparatively narrow confidence intervals further demonstrated the stability of the observed performance improvements. From a modeling perspective, conventional tree-based methods perform piecewise partitioning within a fixed feature space and therefore have limited capacity to represent temporal ordering and cross-modal relationships. TCN enlarges its receptive field through dilated convolution, but shared convolutional kernels are applied across positions, limiting the dynamic characterization of relationships between distant trade nodes. Although Transformer can model global dependencies, device reliability and process legality are not explicitly distinguished. BERT, CLIP, and VisualBERT primarily emphasize textual semantics, global image–text matching, and vision–language interaction, respectively, but none of them fully integrates trajectory, weighing, electronic seal, and environmental sensing evidence. In contrast, TradeSense-EQA suppresses noise introduced by missing observations, sensor drift, and offline devices through reliability-aware weighting, thereby producing fused representations that more closely reflect actual logistics states. Declaration–fact alignment directly compares declared weights, planned routes, and commodity descriptions with their corresponding physical observations. Process-constrained attention further restricts information propagation to valid trade nodes, thereby reducing false detections caused by irrelevant contextual variables. Joint evidence-chain learning also strengthens shared supervision between anomaly classification and node localization, enabling simultaneous improvements in minority-class Recall, class-balanced performance, and overall ranking capability.

### 4.3. English Trade Risk Question-Answering Task

This experiment was designed to compare the factual extraction, semantic generation, and cross-modal reasoning capabilities of different question-answering models in the evidence-grounded English trade risk question-answering task. Particular attention was given to whether accurate responses could be generated from specific trade documents, sensor records, and anomaly evidence chains.

**Figure 5:**
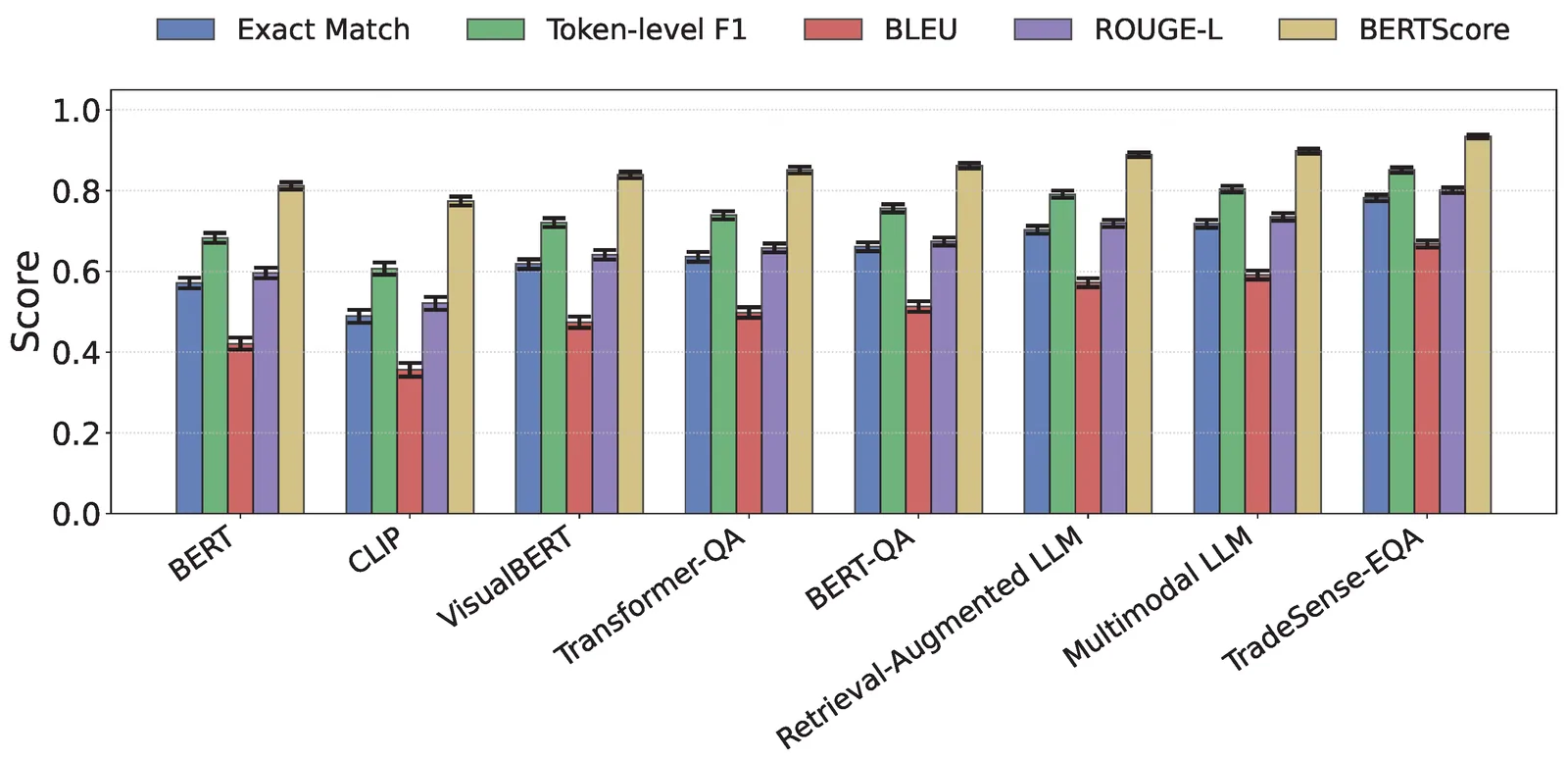
Performance comparison of different methods in terms of Exact Match, Token-level F1, BLEU, ROUGE-L, and BERTScore on the evidence-grounded English trade risk question-answering task, with TradeSense-EQA achieving the best results across all metrics

As shown in Table 3 and Figure 5, BERT achieved an Exact Match of 0.571 and a BERTScore of 0.812, indicating that bidirectional contextual encoding can capture the semantics of trade documents, although a dedicated answer-localization and generation mechanism is absent. CLIP produced the lowest results across all metrics, with an Exact Match of only 0.489, demonstrating that its contrastive learning objective is more suitable for global image–text matching than for fine-grained evidence extraction. By jointly encoding visual regions and textual tokens, VisualBERT increased the Token-level F1 to 0.721, indicating that visual evidence contributed to questions involving cargo conditions and electronic seals. Transformer-QA achieved an Exact Match of 0.636 and a BERTScore of 0.851, reflecting the effectiveness of self-attention in modeling long-range question–evidence relationships. Through the prediction of answer start and end positions, BERT-QA further improved the Exact Match and Token-level F1 to 0.661 and 0.756, respectively, although its support for open-ended risk explanations remained limited. Retrieval-Augmented LLM and Multimodal LLM achieved Exact Match scores of 0.703 and 0.718, respectively, demonstrating that external retrieval and multimodal input substantially improved answer quality. Nevertheless, incorrectly selected evidence could still propagate errors into the generation stage.

TradeSense-EQA achieved the best results across all metrics, with Exact Match, Token-level F1, BLEU, ROUGE-L, and BERTScore values of 0.782, 0.851, 0.668, 0.801, and 0.934, respectively. Its narrower confidence intervals indicated greater stability in answer accuracy, token coverage, linguistic quality, and semantic consistency. From a modeling perspective, BERT and BERT-QA primarily rely on textual sequence representations, and their attention space is largely restricted to documentary context. CLIP adopts a global contrastive objective and may therefore overlook localized evidence such as timestamps, weight values, and device states. Conventional retrieval-augmented models generally select evidence according to the semantic similarity between a question and textual passages, making it difficult to jointly evaluate device reliability and process legality. In TradeSense-EQA, a dual-encoder retriever is first used to reduce the candidate evidence space. Cross-attention is subsequently applied to rerank the candidates by jointly considering evidence source, temporal position, device reliability, and node-level risk. An evidence-permission mask blocks low-relevance or low-confidence information. The combination of vocabulary generation and evidence copying further enables port names, weight values, and timestamps to be directly extracted from original records, thereby reducing the probability that critical facts are altered or nonexistent information is generated. The abstention mechanism and confidence calibration further prevent unsupported answers when the available evidence is insufficient, making the generated results more suitable for high-risk trade review scenarios.

### 4.4. Ablation Results

This experiment was designed to evaluate the individual contributions of the major components of TradeSense-EQA to anomaly detection, evidence-chain construction, and English question answering, while also examining the complementary relationships among these components.

**Figure 6:**
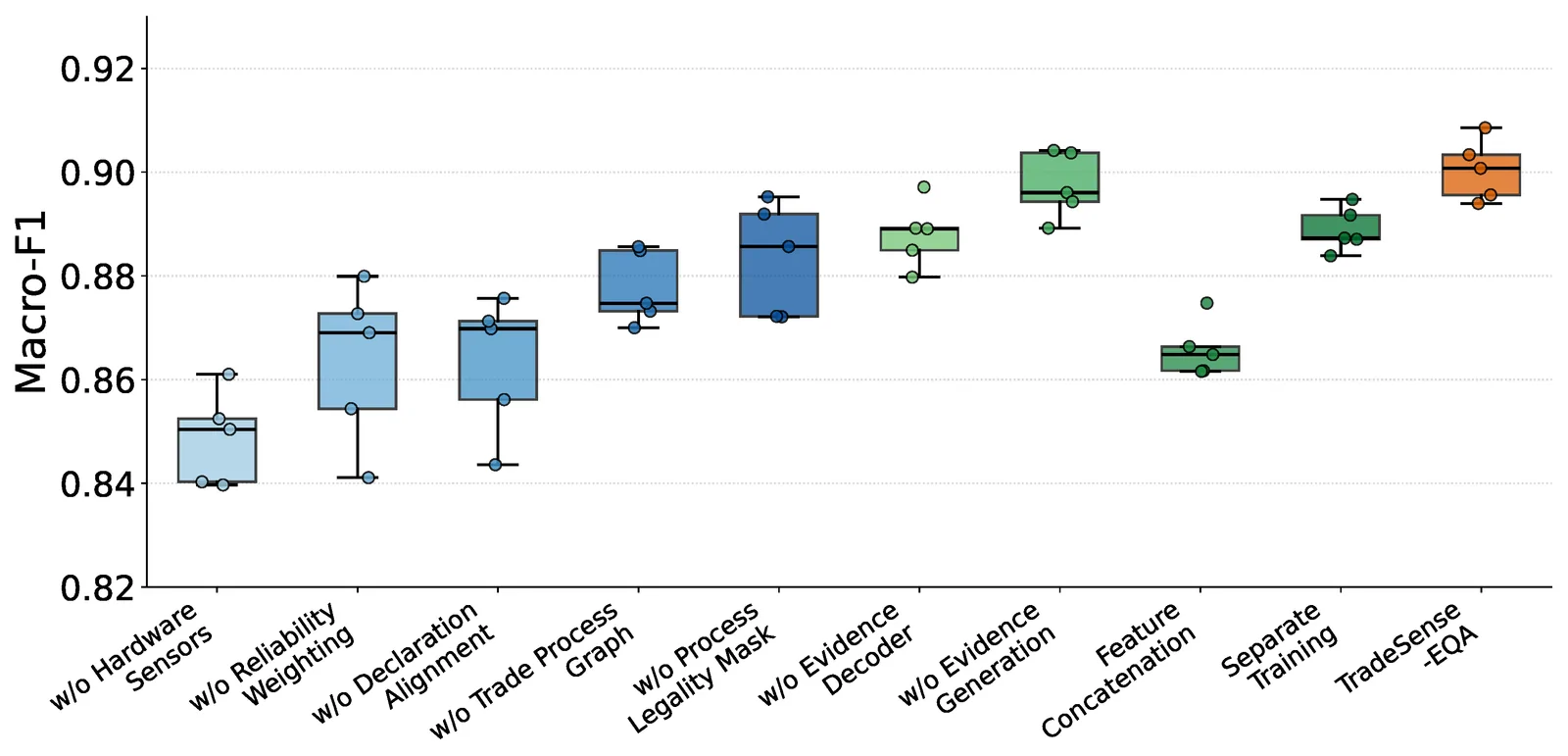
Macro-F1 distributions of TradeSense-EQA and its ablated variants over five independent runs, demonstrating that the complete model provides the highest and comparatively stable anomaly detection performance.

As shown in Table 4 and Figure 6, the complete model achieved a Macro-F1 of 0.903, a ROC-AUC of 0.958, an Evidence F1 of 0.824, an Exact Match of 0.782, and a BERTScore of 0.934. The relatively narrow confidence intervals across these metrics indicated the stability of the unified framework. After hardware sensor data were removed, the Macro-F1 decreased to 0.842 and the Evidence F1 decreased to 0.693, indicating that actual logistics states cannot be reliably verified using only trade documents and structured declaration fields. Without reliability-aware weighting, the Macro-F1 and ROC-AUC decreased to 0.868 and 0.927, respectively, demonstrating that sensor noise, missed readings, and device disconnections adversely affect multisource fusion. When declaration–fact alignment was removed, the Macro-F1 decreased to 0.859 and the Evidence F1 decreased to 0.704, confirming that explicit comparison between digital declarations and physical observations is essential for identifying trade authenticity anomalies. After removing the trade-process graph and process-legality mask, the Evidence F1 decreased to 0.748 and 0.759, respectively, indicating that the absence of business-order constraints weakens the modeling of relationships among anomalous nodes. Removing the evidence-chain decoder produced the lowest Evidence F1 of 0.681, demonstrating that node-level classification results cannot automatically form complete evidence paths. When evidence-constrained generation was removed, the classification metrics changed only slightly, whereas the Exact Match and BERTScore decreased to 0.703 and 0.884, respectively, confirming that this component primarily affects factual answer accuracy and semantic consistency. Simple feature concatenation and separate task training also produced substantially lower overall performance than the complete model.

From a modeling perspective, hardware sensors provide physical observations that are independent of documentary descriptions, allowing latent trade states to be estimated from multiple evidence sources. Their removal therefore substantially increases representation uncertainty. Reliability-aware weighting reduces the contribution of noisy sensing channels through normalized gating. Under the conditional error-ordering assumption derived in Section 3.3.2, this redistribution tightens the upper bound of the fused representation error; outside that condition, the ablation result supplies empirical rather than universal support for robustness. In contrast, fixed weighting and simple feature concatenation implicitly assume equal reliability across modalities and may amplify deviations generated by faulty devices. Declaration–fact alignment projects declared weights, routes, and commodity information together with their corresponding sensor records into a shared representation space, enabling cross-modal discrepancies to be learned directly rather than inferred from indirect correlations. The trade-process graph and process-legality mask restrict attention propagation to nodes that satisfy temporal and business relationships, reducing the probability that unrelated nodes are combined into spurious evidence chains. The evidence-chain decoder preserves continuous and mutually consistent anomalous nodes through global path optimization and therefore has the greatest effect on Evidence F1. Evidence-constrained generation employs restricted attention and evidence-copying mechanisms to ensure that critical values, timestamps, and device events are directly derived from retrieved evidence. Multitask joint training shares supervision across anomaly classification, node localization, and question answering, allowing the underlying representations to be simultaneously constrained by detection and semantic objectives. Consequently, the unified model consistently outperformed separately trained task-specific models. The ablation result also clarifies the role of the large-language-model decoder. Removing evidence-constrained generation changes Macro-F1 from 0.903 to 0.899, a difference of only 0.004 and smaller than the reported uncertainty intervals; this should not be interpreted as a meaningful anomaly-detection gain. The decoder is retained for evidence-grounded answers, where the same ablation reduces Exact Match from 0.782 to 0.703 and BERTScore from 0.934 to 0.884. The detection and evidence-chain branches can therefore be deployed without the generative decoder when only risk scoring is required, while LLM generation is invoked asynchronously for analyst queries or flagged cases. This modular operating mode avoids placing the decoder on the latency-critical detection path and makes the computational trade-off explicit.

### 4.5. Computational Overhead Analysis

In addition to detection accuracy, computational efficiency is an important consideration for the practical deployment of cross-border trade risk monitoring systems. This experiment was therefore designed to evaluate the computational overhead of TradeSense-EQA and representative deep learning and multimodal baseline models under the same hardware and software environment described in Section 4.1.1. The number of trainable parameters and floating-point operations (FLOPs) were used to characterize model size and computational complexity, respectively. Peak GPU memory consumption during inference was recorded to evaluate hardware-resource requirements, while average inference latency and processing throughput were measured to assess runtime efficiency. All models were evaluated using the same test protocol, and latency was calculated as the average processing time per trade sample after model warm-up. Throughput was measured as the number of trade samples processed per second. These measurements jointly characterize the trade-off between predictive capability and computational cost and provide a practical assessment of whether the proposed framework can support large-scale cross-border trade risk screening and intelligent port monitoring.

As shown in Table 5, TradeSense-EQA incurs a higher computational cost than the unimodal and lightweight temporal baselines because it jointly performs multimodal encoding, reliability-aware fusion, declaration–fact alignment, and process-constrained evidence reasoning. TCN achieved the lowest computational overhead, requiring only 4.2 M parameters, 2.8 G FLOPs, and 1.3 GB of peak GPU memory, with an average latency of 6.8 ms per sample. In comparison, multimodal models such as MulT and Perceiver IO required substantially greater computation and memory, reflecting the additional cost of jointly processing heterogeneous inputs. TradeSense-EQA contained 142.7 M trainable parameters and required 36.9 G FLOPs, with a peak GPU memory consumption of 6.8 GB. Its average inference latency was 38.9 ms per sample, corresponding to a throughput of 25.7 samples/s. Although TradeSense-EQA is computationally more demanding than the baseline models, the observed overhead remains within a practical range for GPU-based deployment. In particular, its memory consumption remains well below the available GPU capacity of the experimental platform, while the measured throughput allows multiple trade events to be screened per second. Considering the performance gains reported in Table 2, the additional computational cost represents a trade-off for improved anomaly discrimination, cross-modal consistency verification, and evidence-chain construction. Moreover, the anomaly-detection branch can operate independently of the evidence-grounded language-model decoder, allowing risk screening to remain on the latency-critical path while question answering is invoked asynchronously only for flagged cases or analyst queries. This modular deployment strategy further reduces the practical impact of the additional computational overhead.

### 4.6. Discussion

The experimental results demonstrate that the practical value of TradeSense-EQA extends beyond improvements in cross-border trade anomaly classification accuracy and further supports cross-border trade and financial risk assessment. More importantly, digital declarations in trade documents are directly connected with physical facts observed throughout cargo transportation. During port-entry and pre-loading inspections, regulatory personnel are generally required to jointly examine declared weights, quantities listed in packing documents, weighbridge records, RFID readings, and container images. These data are usually presented in separate systems, leaving anomaly identification dependent on manual comparison. TradeSense-EQA directly aligns declared weights with port weighing results and combines cargo images, RFID-recorded quantities, and electronic seal states to determine whether an observed discrepancy is attributable to routine handling operations, device error, or an actual change in cargo condition. For example, when a substantial difference is detected between the declared weight and the departure-port weighing record, while insufficient RFID readings and an abnormal loading distribution are simultaneously observed in the container image, these dispersed observations can be organized into a unified risk path. High-risk containers can therefore be prioritized for customs inspection rather than being flagged solely according to an isolated threshold violation.

In transportation and transshipment scenarios, GPS and AIS trajectories may be affected by signal obstruction, positioning drift, and communication interruptions, while electronic seals may also experience short-term disconnections. The reliability-aware mechanism combines device health states, gateway logs, and observations from neighboring sensors to reduce false alarms caused by technical failures. For example, a vehicle stop accompanied by a seal-opening event within an authorized inspection zone may represent a legitimate inspection. In contrast, a seal-opening event at an unauthorized location, combined with route deviation, abnormal stopping behavior, and reduced arrival weight, can be identified as a composite risk supported by continuous evidence. In cold-chain trade, a short-term temperature fluctuation may result from routine door opening during loading or unloading. However, a persistent temperature increase accompanied by changes in the container door state, transportation delays, and abnormal vibration is more likely to indicate cold-chain interruption or improper cargo handling. The model outputs can therefore assist ports, logistics enterprises, and insurance institutions in distinguishing device failures, legitimate operations, and genuine transportation risks and their associated financial risks.

Evidence-grounded English question answering further improves the applicability of the framework in international trade collaboration. Customs officers, logistics managers, and financial institutions conducting trade-background reviews can directly ask about the stage at which an anomaly occurred, the actual weighing result, the location of a seal-opening event, or the evidence supporting a risk judgment. Corresponding responses are generated by citing specific documentary fields, sensor records, and timestamps. When payment amounts, declared trade values, and physical cargo conditions are simultaneously inconsistent, the model can indicate that additional verification of trade authenticity, financial settlement consistency, and fund flows is required. However, a transaction is not directly classified as fraudulent trade or trade-based money laundering when the available evidence is insufficient. Anomaly detection outputs are therefore transformed into readable, traceable, and reviewable business evidence, providing operational support for customs inspection prioritization, port security management, logistics liability determination, trade-finance review, and cross-border compliance investigations.

### 4.7. Limitations and Future Work

Several limitations remain. The current dataset was primarily collected from Tianjin Port, Ningbo-Zhoushan Port, and Yantai Port. Although conventional container transportation, cold-chain logistics, and port handling operations were included, substantial differences exist across countries in regulatory systems, trade rules, device deployment strategies, and commodity structures. The generalization capability of the model to other ports, cross-border road transportation, and air freight therefore requires further validation. Some anomaly categories occur infrequently, and only a limited number of composite fraud cases and judicially confirmed trade-based money laundering cases were available, which may affect the stability of minority high-risk category identification. Heterogeneous sensing devices may also differ in model specifications, calibration offsets, long-term disconnection patterns, and data interfaces. The current reliability assessment mainly relies on device states and historical quality information and therefore cannot completely exclude deliberate manipulation or simultaneous failures of multiple sensors. In addition, the generated evidence chains represent associations constrained by business processes and should not be directly interpreted as strict causal relationships. English question-answering results still depend on sequential processing across speech recognition, evidence retrieval, and large language model generation, allowing upstream errors to propagate to the final answer. Future research should expand the dataset across countries, ports, and transportation modes. Federated learning and privacy-preserving computation could be incorporated to support collaborative model training across multiple institutions. Structural causal models, online sensor calibration, and feedback from human review could also be integrated to improve adaptation to emerging anomalies, device attacks, and complex cross-border trade risks.

## 5. Conclusions

To address the challenges associated with the unified verification of digital declarations and actual logistics processes in cross-border trade, substantial variations in hardware sensor quality, and the limited traceability of risk assessment results, TradeSense-EQA was proposed as a cross-border trade security anomaly detection and evidence-grounded English question-answering framework. Centered on artificial intelligence-driven sensing, the proposed framework maps trade documents, structured declaration fields, financial settlement records, GPS/AIS trajectories, RFID records, electronic seal events, port weighing data, temperature and humidity measurements, vibration signals, container door states, and visual images into a unified trade-process representation, thereby enabling joint verification of digital declarations and physical facts. The reliability-aware multisource trade sensing representation module dynamically adjusts information weights according to missing-data conditions, device health states, communication quality, and support from neighboring sensors, reducing the influence of missed readings, sensor drift, and short-term disconnections on risk assessment. The trade-process-constrained evidence-chain anomaly detection module identifies composite anomalies across declaration, packing, transportation, transshipment, arrival, and customs clearance stages and constructs temporally ordered evidence paths supported by business-process relationships. The evidence-grounded English question-answering module generates risk-oriented responses from verified documentary fields and sensor records, while confidence estimation and abstention mechanisms are employed to reduce unsupported generation. Experimental results demonstrated that TradeSense-EQA achieved an Accuracy of 0.918, a Precision of 0.909, a Recall of 0.897, a Macro-F1 of 0.903, and a ROC-AUC of 0.958 on the cross-border trade anomaly detection task, consistently outperforming conventional machine learning, temporal modeling, and multimodal baseline methods. On the English trade risk question-answering task, Exact Match, Token-level F1, ROUGE-L, and BERTScore reached 0.782, 0.851, 0.801, and 0.934, respectively. The ablation results further confirmed the independent contributions of hardware sensing data, reliability-aware weighting, declaration–fact alignment, trade-process graph modeling, and evidence-constrained generation. Overall, intelligent sensing, multimodal learning, process-aware evidence reasoning, and large language model-based question answering were integrated within a unified framework. The resulting system provides more reliable, interpretable, and auditable technical support for customs supervision, port security management, international logistics review, financial risk review, and trade-background investigation.

## Figures and Tables

**Table 1:** Composition of the cross-border trade security anomaly detection dataset and data acquisition devices.

Data Type	Hardware Sensor/Acquisition Device	Data Volume
Trade document text	Ricoh fi-8170 document scanner (PFU Limited, Kahoku, Japan)	128,460 records
Structured declaration fields	Trade information management system deployed on a Dell PowerEdge R740 server (Dell Technologies Inc., Round Rock, TX, USA)	86,320 records
GPS transportation trajectories	Queclink GV300 series positioning terminal (Queclink Wireless Solutions Co., Ltd., Shanghai, China)	42,680 records
AIS vessel trajectories	Furuno FA-170 Automatic Identification System (AIS) transponder (Furuno Electric Co., Ltd., Nishinomiya, Japan)	31,900 records
RFID node records	Impinj R700 RFID reader (Impinj, Inc., Seattle, WA, USA)	28,450 records
Port weighing data	Mettler Toledo IND570 industrial weighing terminal (Mettler-Toledo AG, Greifensee, Switzerland)	39,760 records
Temperature and humidity data	Sensirion SHT35 temperature and humidity sensor (Sensirion AG, Stäfa, Switzerland)	35,720 sequences
Vibration and impact data	Bosch BMI160 inertial sensor (Bosch Sensortec GmbH, Reutlingen, Germany)	16,850 records
Electronic seal states	Savi Secure ST-900-CV electronic security device (Savi Technology, Alexandria, VA, USA)	29,140 records
Port visual images	Hikvision MV-CA050-20GC industrial camera (Hangzhou Hikvision Digital Technology Co., Ltd., Hangzhou, China)	64,200 images
X-ray inspection images	NUCTECH MB1215DE container/vehicle X-ray inspection system (Nuctech Company Limited, Beijing, China)	18,600 images
English speech queries	Shure MV7 noise-reduction microphone (Shure Incorporated, Niles, IL, USA)	12,500 clips
Auxiliary settlement records	Enterprise payment management system deployed on an IBM Power System S922 server (International Business Machines Corporation, Armonk, NY, USA)	51,600 records

**Table 2:** Performance comparison of different methods on the cross-border trade security anomaly detection task. Results are reported as the mean ± 95% confidence interval over five independent runs. MulT and Perceiver IO are evaluated using the same complete multimodal input set as TradeSense-EQA. Bold values indicate the best performance achieved among all compared methods for each evaluation metric.

Method	Accuracy ↑	Precision ↑	Recall ↑	Macro-F1 ↑	ROC-AUC ↑
XGBoost	0.842±0.009	0.831±0.011	0.806±0.014	0.817±0.012	0.884±0.008
LightGBM	0.851±0.008	0.840±0.010	0.818±0.013	0.828±0.011	0.892±0.007
TCN	0.866±0.008	0.854±0.009	0.839±0.011	0.846±0.010	0.907±0.007
Transformer	0.879±0.007	0.868±0.009	0.851±0.010	0.859±0.009	0.918±0.006
BERT	0.858±0.009	0.849±0.010	0.824±0.013	0.836±0.011	0.899±0.008
CLIP	0.845±0.010	0.836±0.012	0.811±0.014	0.823±0.013	0.890±0.009
VisualBERT	0.884±0.007	0.873±0.008	0.860±0.010	0.866±0.008	0.923±0.006
BERT-QA	0.861±0.008	0.850±0.010	0.832±0.012	0.841±0.010	0.902±0.007
MulT	0.897±0.006	0.887±0.008	0.875±0.009	0.881±0.008	0.941±0.005
Perceiver IO	0.904±0.006	0.895±0.007	0.883±0.008	0.889±0.007	0.947±0.005
TradeSense-EQA	0.918±0.005	0.909±0.006	0.897±0.007	0.903±0.006	0.958±0.004

**Table 3:** Performance comparison of different question-answering methods on the evidence-grounded English trade risk question-answering task. Results are reported as the mean ± 95% confidence interval over five independent runs. Bold values indicate the best performance achieved among all compared methods for each evaluation metric.

Method	Exact Match ↑	Token-Level F1 ↑	BLEU ↑	ROUGE-L ↑	BERTScore ↑
BERT	0.571±0.013	0.683±0.012	0.421±0.015	0.596±0.013	0.812±0.009
CLIP	0.489±0.016	0.607±0.015	0.356±0.017	0.521±0.016	0.774±0.011
VisualBERT	0.618±0.012	0.721±0.011	0.474±0.014	0.641±0.012	0.839±0.008
Transformer-QA	0.636±0.012	0.739±0.010	0.498±0.013	0.658±0.011	0.851±0.008
BERT-QA	0.661±0.011	0.756±0.010	0.513±0.013	0.674±0.010	0.862±0.007
Retrieval-Augmented LLM	0.703±0.010	0.791±0.009	0.572±0.011	0.719±0.009	0.889±0.006
Multimodal LLM	0.718±0.010	0.804±0.008	0.591±0.011	0.735±0.009	0.898±0.006
TradeSense-EQA	0.782±0.008	0.851±0.007	0.668±0.009	0.801±0.007	0.934±0.004

**Table 4:** Ablation results for the main components of TradeSense-EQA. Results are reported as the mean ± 95% confidence interval over five independent runs. Bold values indicate the best performance achieved among all compared methods for each evaluation metric.

Model Variant	Macro-F1 ↑	ROC-AUC ↑	Evidence F1 ↑	Exact Match ↑	BERTScore ↑
w/o Hardware Sensors	0.842±0.010	0.904±0.007	0.693±0.012	0.721±0.011	0.892±0.007
w/o Reliability-Aware Weighting	0.868±0.009	0.927±0.006	0.731±0.011	0.742±0.010	0.906±0.006
w/o Declaration–Fact Alignment	0.859±0.009	0.918±0.007	0.704±0.012	0.728±0.011	0.897±0.007
w/o Trade Process Graph	0.877±0.008	0.934±0.006	0.748±0.010	0.751±0.010	0.911±0.006
w/o Process Legality Mask	0.884±0.007	0.940±0.005	0.759±0.010	0.759±0.009	0.915±0.006
w/o Evidence-Chain Decoder	0.892±0.007	0.946±0.005	0.681±0.013	0.736±0.011	0.901±0.007
w/o Evidence-Constrained Generation	0.899±0.006	0.951±0.004	0.792±0.009	0.703±0.012	0.884±0.008
Simple Feature Concatenation	0.863±0.009	0.922±0.006	0.716±0.011	0.733±0.010	0.900±0.007
Separate Task Training	0.887±0.007	0.942±0.005	0.771±0.009	0.764±0.009	0.918±0.005
TradeSense-EQA	0.903±0.006	0.958±0.004	0.824±0.008	0.782±0.008	0.934±0.004

**Table 5:** Computational overhead comparison of different deep learning and multimodal methods. All methods were evaluated under the same hardware and software environment.

Method	Parameters (M) ↓	FLOPs (G) ↓	Peak GPU Memory (GB) ↓	Inference Latency (ms/Sample) ↓	Throughput (Samples/s) ↑
TCN	4.2	2.8	1.3	6.8	147.1
Transformer	18.9	8.6	2.1	10.9	91.7
BERT	109.5	22.4	3.4	18.7	53.5
CLIP	151.3	18.7	4.7	24.8	40.3
VisualBERT	116.8	27.9	5.2	30.6	32.7
BERT-QA	109.5	22.6	3.6	20.4	49.0
MulT	47.6	31.8	5.9	34.5	29.0
Perceiver IO	63.4	28.5	5.5	31.2	32.1
TradeSense-EQA	142.7	36.9	6.8	38.9	25.7

## Data Availability

The data presented in this study are available on request from the corresponding author.
